# Biogeographic problem-solving reveals the Late Pleistocene translocation of a short-faced bear to the California Channel Islands

**DOI:** 10.1038/s41598-020-71572-z

**Published:** 2020-09-16

**Authors:** Alexis M. Mychajliw, Torben C. Rick, Nihan D. Dagtas, Jon M. Erlandson, Brendan J. Culleton, Douglas J. Kennett, Michael Buckley, Courtney A. Hofman

**Affiliations:** 1 Department of Anthropology, University of Oklahoma, Norman, OK USA; 2 Laboratories of Molecular Anthropology and Microbiome Research, University of Oklahoma, Norman, OK USA; 3 La Brea Tar Pits and Museum, Los Angeles, CA USA; 4 Department of Anthropology, National Museum of Natural History, Smithsonian Institution, Washington, DC USA; 5 Museum of Natural and Cultural History, University of Oregon, Eugene, OR USA; 6 Department of Anthropology, University of Oregon, Eugene, OR USA; 7 Institutes of Energy and the Environment, The Pennsylvania State University, University Park, PA USA; 8 Department of Anthropology, University of California, Santa Barbara, CA USA; 9 School of Natural Sciences, Manchester Institute of Biotechnology, University of Manchester, Manchester, M1 7DN UK

**Keywords:** Biogeography, Palaeoecology, Palaeoecology, Archaeology

## Abstract

An accurate understanding of biodiversity of the past is critical for contextualizing biodiversity patterns and trends in the present. Emerging techniques are refining our ability to decipher otherwise cryptic human-mediated species translocations across the Quaternary, yet these techniques are often used in isolation, rather than part of an interdisciplinary hypothesis-testing toolkit, limiting their scope and application. Here we illustrate the use of such an integrative approach and report the occurrence of North America’s largest terrestrial mammalian carnivore, the short-faced bear, *Arctodus simus,* from Daisy Cave (CA-SMI-261), an important early human occupation site on the California Channel Islands. We identified the specimen by corroborating morphological, protein, and mitogenomic lines of evidence, and evaluated the potential natural and anthropogenic mechanisms of its transport and deposition. While representing just a single specimen, our combination of techniques opened a window into the behavior of an enigmatic species, suggesting that *A. simus* was a wide-ranging scavenger utilizing terrestrial and marine carcasses. This discovery highlights the utility of bridging archaeological and paleontological datasets to disentangle complex biogeographic scenarios and reveal unexpected biodiversity for island systems worldwide.

## Introduction

Islands have long been important places for evaluating fundamental ecological and evolutionary processes and are areas of significant conservation concern^1^. Yet, we are increasingly recognizing that the structures of today’s island ecosystems are partly a result of human activities in the historical and more distant past, both through selective extinctions^2^ and sometimes cryptic species introductions^3^. Fossil and archaeological records can play a critical role in disentangling natural versus anthropogenically-mediated dispersals with the application of emerging technical toolkits (e.g., ancient DNA, stable isotopes). Even when radiocarbon chronologies suggest a pre- or post- anthropogenic context, discerning whether the rare presence of a species—such as an isolated skeletal element—is due to a live arrival or post-mortem transport is challenging, yet this distinction has significant consequences for understanding past ecosystem change^4^. Solving such ambiguous cases requires a robust hypothesis-testing framework that considers taphonomic, geologic, ecological, and archaeological contexts, as well as appropriate knowledge of the biology and native range of the species in question.

**Figure 1:**
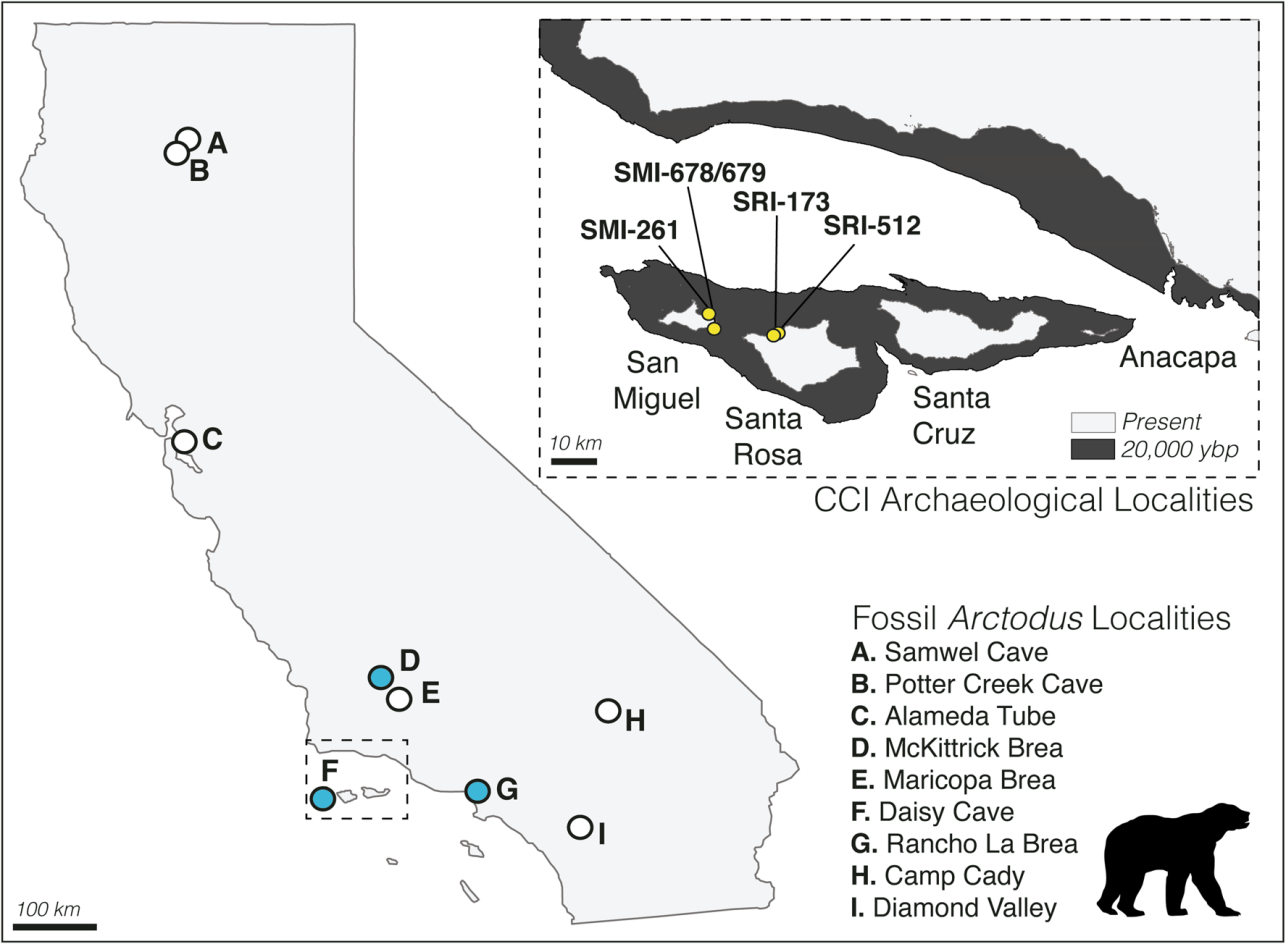
The Late Quaternary range of the short-faced bear, *Arctodus simus*, in California. Locations of Late Pleistocene (approximately Rancholabrean) *Arctodus simus* occurrences: white circles indicate presence at a site of indirectly estimated age^75–79^ and blue circles indicate directly radiocarbon dated specimens^27,59^. Inset map features the California Channel Islands with coastlines of both the present day and ~ 20,000 years before present^6^. Yellow circles depict pertinent archaeological sites. Map produced using the statistical software R v3.6.2 and Adobe Illustrator.

California’s Channel Islands (CCI) are an ideal system to refine our diagnostic toolkit due to the well-studied geologic and human histories and communities of iconic endemic fauna. The CCI consist of eight islands that are currently located approximately 20–100 km from the California coastline. The size of the islands and their distance to the mainland has shifted with past glacial-interglacial cycles, but they were never connected by a land or ice bridge during the Quaternary (Fig. 1). The CCI are split into two north–south island groupings with distinct geologic histories: the northern islands (Santa Rosa, San Miguel, Santa Cruz, Anacapa) were joined as a single superisland, Santarosae, as recently as 10,000 years ago, whereas the southern islands have always been more isolated^5,6^ (Fig. 1). Given these dispersal filters and fluctuations in sea level, the CCI terrestrial faunal communities are depauperate compared to the nearby mainland, with a total of just 10 known extinct and extant terrestrial mammals^7^. This biogeographic history provides a hypothetical timeline for the accumulation and loss of fauna through natural dispersal events; glacial lowstands allowed for the recurrent colonization of Santarosae by Columbian mammoths (*Mammuthus columbianus*), which swam to the island and evolved into the endemic Channel Islands mammoth (*Mammuthus exilis*)^5^.

**Figure 2:**
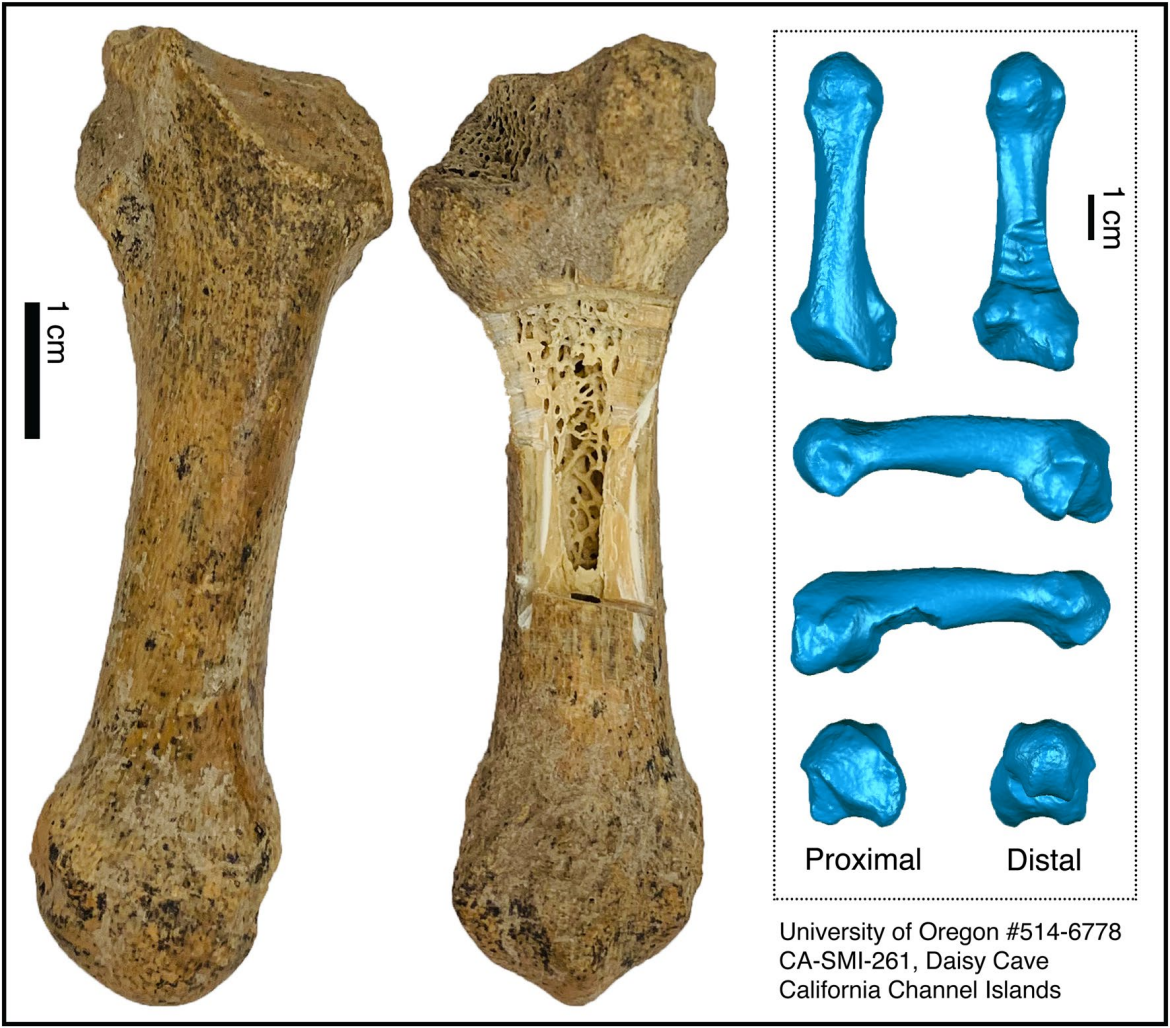
Photographs and 3D models of the *Arctodus simus* specimen from Daisy Cave. Scans were created using a structured light DAVID SLS3-3D scanner and Geomagic (Supplemental Data File 1). Photographs were taken by the authors.

Humans colonized Santarosae ~ 13,000 years ago, although there are hints of a possible earlier human presence^8,9^. The island’s more than 700 archaeological sites document substantial anthropogenic influences on the ecology of the islands from the Late Pleistocene to the present^7^, including the introduction of taxa previously considered to be native endemic lineages. Daisy Cave (CA-SMI-261), a well-studied and finely stratified archaeological and paleontological site on San Miguel Island^8^, has yielded a diverse assemblage of faunal remains that provide critical insights about the relationships between Indigenous peoples and the endemic fauna of the CCI^10,11^. For example, the island fox *(Urocyon littoralis*)*–* a charismatic species of significant conservation concern– may have been introduced by humans between ~ 7 and 9,000 years ago^12^, and data increasingly support an anthropogenically-mediated translocation of the island deer mouse, *Peromyscus maniculatus*, ~ 10,000 years ago^13^. Here we report a new addition to the biodiversity register of Daisy Cave: a large metapodial recovered during excavations in 1993 (Fig. 2), which we identify as the short-faced bear, *Arctodus simus* (Carnivora: Ursidae: Tremarctinae^14^), based on morphology, ancient DNA, and collagen fingerprinting (zooarchaeological mass spectrometry, ZooMS^15^). These data help address longstanding questions about the ecology and biogeography of this enigmatic carnivore south of Canada.

The short-faced bear was the largest carnivorous mammal native to North America, weighing 700–1,000 kg^16^. It is known from more than 100 Late Quaternary localities across a wide biogeographic range spanning Beringia to Mexico, California, and Florida^17^. Decades of debate have centered on the diet and life history of this enigmatic species; morphological diagnoses have included potential feeding ecologies as a hypercarnivore^14^, kleptoparasite^18^, omnivore^16^, and herbivore^19^. While stable isotopes from Beringian populations point to a carnivorous diet^18,20,21^, more indirect diet proxies such as dental caries^22^ and dental microwear^23^ dispute scavenging and high meat consumption in California populations. We provide the first collagen stable isotope values for this species in lower latitudes to address this gap in basic natural history knowledge of relevance to Late Quaternary extinctions and inform hypothetical mechanisms of dispersal to the CCI. We present four hypotheses to explain the presence of this megafaunal carnivore on the CCI by either pre or post-mortem transport: (1) a population of *A. simus* existed on the CCI following dispersals during glacial lowstands; (2) a single *A. simus* individual dispersed to the CCI; (3) humans or 4) a bird of prey carried the metapodial from the mainland and deposited it in Daisy Cave (see Supplemental Table 1 for hypothesis-specific expectations). Our approach is widely applicable to other island systems and unraveling ambiguous biogeographic distributions globally.

**Table 1:** Radiocarbon date and stable isotope values of the CCI *A. simus* metacarpal.

Lab ID	^14^C age	Mean cal ybp	σ cal ybp	Median cal ybp	95.4% Range	%C	%N	C:N	Collagen yield	δ^13^C‰	δ^15^N‰
UCIAMS-137939; PSU-5973	14,130 ± 70	17,009	135	17,016	16,713–17,296	46.9	17.4	3.15	11.9% crude/6.9% UF*	-17.9	13.2

*UF = ultrafiltered collagen yield. The radiocarbon date was calibrated with a mixed curve 20% MarineCal13, 80% IntCal1382, with a Delta_R = 225 ± 35 ^44^, using OxCal v 4.345. δ^13^C is ‰ VPDB and δ^15^N is ‰ atm N2.

## Results

### Morphological assessment

Systematic Paleontology.

Class Mammalia Linnaeus, 1758.

Order Carnivora Bowdich, 1821.

Family Ursidae Gray, 1825.

Subfamily Tremarctinae Kraglievich, 1926.

Genus *Arctodus* Leidy, 1854.

*Arctodus simus* (Cope), 1879.

#### Referred material

Left metacarpal I (University of Oregon #514–6,778), San Miguel Island, California Channel Islands, USA (Fig. 2).

#### Description

The specimen is a left metacarpal I. It has external abrasions, possibly resulting from screening of site sediments, but has well-preserved internal structures including collagen, as evidenced by a crude collagen yield of 11.9% by weight. The proximal articular surface is damaged. The specimen exhibits fused epiphyses; metacarpal epiphyseal fusion occurs at ages 1–2 years in both sexes *U. americanus*^24^. Using digital calipers, we followed *Arctodus* metapodial measurement guidelines^14^ to measure greatest length (71.03 mm), diameter of proximal end (24.33 mm, approximate), least width of shaft (11.12 mm) and greatest breadth of distal end (17.62 mm), and compared these with *A. simus* metapodial values from the published literature, most notably individuals from nearby Rancho La Brea as well as available measurements for *U. americanus* and *U. arctos* (Supplemental Table 2).

#### Diagnosis

*A. simus* can be distinguished from other California bears by its exceptionally large size. Further, there is currently no evidence for the presence of *U. arctos* in the Late Pleistocene of Southern California; the earliest radiocarbon date available for *U. arctos* in California is from the Middle Holocene (Rancho La Brea, Los Angeles, QC916R, 5,270 ± 155 uncalibrated ^14^C years)^25–27^, but the black bear, *U. americanus*, was widespread. The Daisy Cave specimen compares favorably with a left *A. simus* metacarpal I from Rancho La Brea (LACM Z93).

#### Locality and age

The specimen was recovered along the dripline of the outer rock shelter at Daisy Cave (CA-SMI-261) on San Miguel Island, area/unit: D6 SW ½ × 1, Stratum: I (111 cm depth) (Supplemental Fig. 1). Today Daisy Cave is located on the northeastern coastline of San Miguel Island, but during the Late Pleistocene it was an inland locality at the base of a steep escarpment on northwest Santarosae. Daisy Cave is of both archaeological and paleontological significance, with archaeological shell midden deposits spanning the terminal Pleistocene and Holocene and deeper layers containing mice and other animal remains dated to the Late Pleistocene with evidence of raptor activity^8,13,28^. Bone collagen extracted from the specimen dates to an estimated calendar age of ~ 17,000 cal ybp (Table 1). Charcoal from Strata I was dated 13,330–13,010 cal ybp (CAMS-9096) and a charred twig fragment from the underlying Stratum J to 13,830–13,490 cal ybp (CAMS-14369) (Supplemental Fig. 1).

### ZooMS collagen fingerprinting

**Figure 3:**
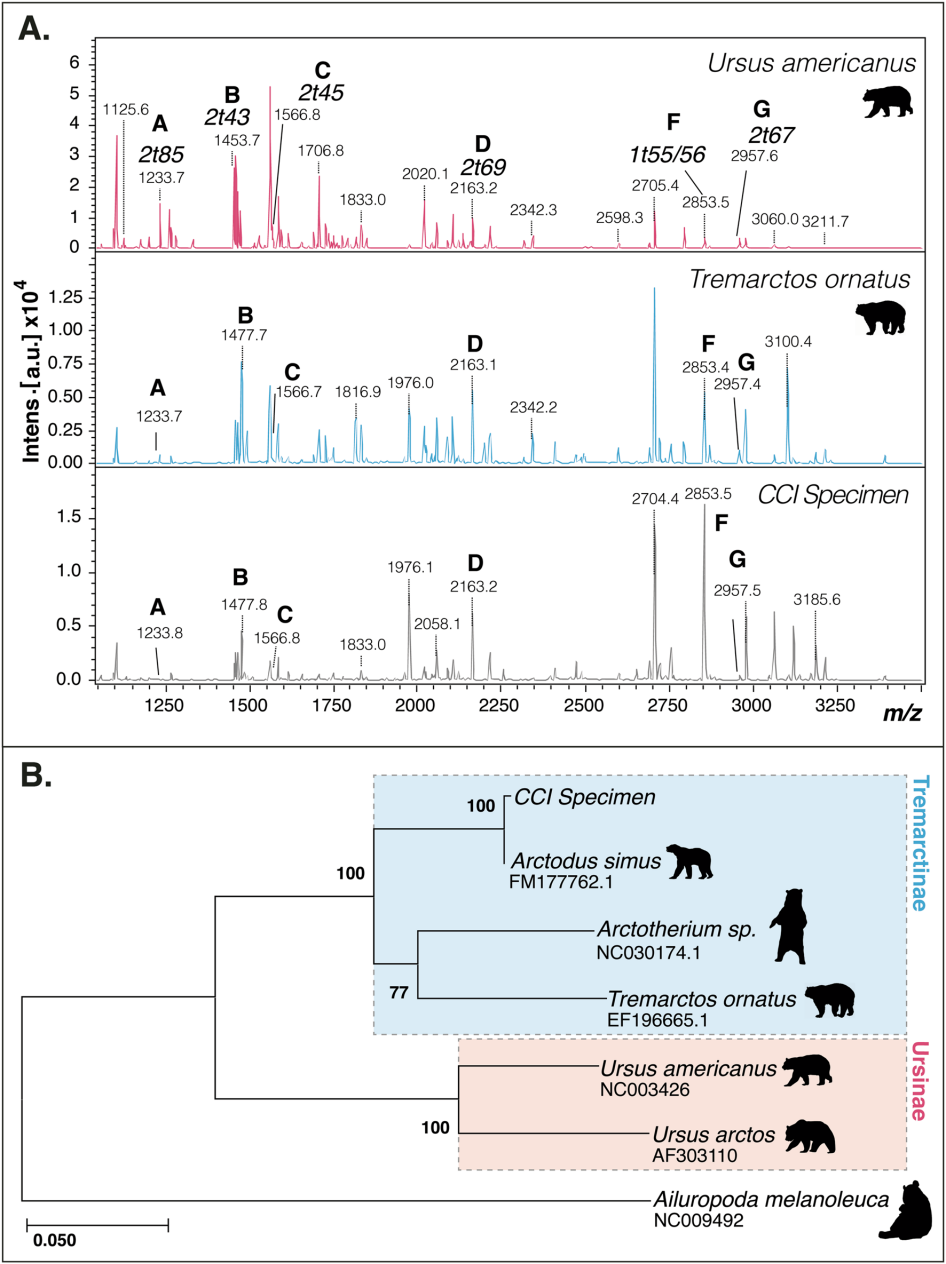
(**A**) Collagen peptide mass fingerprints of brown bear, *U. arctos* (top), spectacled bear, *T. ornatus* (middle) and the CCI specimen (bottom). Sequences are annotated with peptide labels relating to their position in the α chains (1 = α1(I), 2 = α2(I); t = trypsin, the number following is the consecutive peptide number assuming cleavage at K and/or R residues; A: 2t85, B: 2t43, C: 2t45, D: 2t69, F: 1t55/56, G: 2t67)^80^. (**B**) Maximum likelihood partial deletion (90%) phylogeny of six bear mitogenomes produced using MEGA X^72,73^. Numbers at nodes represent percentage of trees from 1,000 bootstrap replicates. The tree is drawn to scale, with branch lengths measured in the number of substitutions per site. Figures were produced using Geneious v. 10.0.8 and Adobe Illustrator.

Acid-solubilised and ultrafiltered collagen was analysed by ZooMS^29^. Although several of the expected peptide biomarkers for bears were observed (e.g., peaks at *m/z* 1,233 and *m/z* 2,163), one of the most abundant ursine markers (*m/z* 1,453) was clearly distinct at *m/z* 1,477 (Fig. 3A). We compared the resultant peptide mass fingerprint with those obtained from spectacled bear (*Tremarctos ornatus*) and *U. americanus*, thus confirming this specimen as tremarctine in origin.

### Mitogenomic sequencing and phylogenetic analysis

Using in-solution sequence hybridization, we successfully sequenced a low coverage mitochondrial genome (0.4x) from the Daisy Cave specimen. We compared the sequence to available data for humans and sequences from *U. americanus*^30^, *U. arctos*^30^, and *T. ornatus*^31^, and the extinct giant bears *A. simus*^32^ and *Arctotherium*^33^. Across 5,575 base pairs of sequence, the CCI bone differed at 11 sites (6 transitions and 5 transversions) from the *A. simus* reference sequence and matched at 99.8% similarity. In comparison, *Arctotherium* shares 96.4% similarity, 93.9% for *T. ornatus*, 92.1% for *U. arctos*, and 91.5% for *U. americanus*. Less than 0.01% of reads mapped to the human mitochondrial genome, indicating low levels of human contamination. Phylogenetic reconstruction using a maximum likelihood framework with partial deletion (90%) also placed this bone with *A. simus* (Fig. 3B). This specimen thus represents the second known mitogenome for the species *A. simus* and provides a contemporaneous geographic contrast with the only other available mitogenome (from Eldorado Creek, Canada^32^).

### Stable isotope analysis

Though insight is limited to a single individual, and should therefore be interpreted cautiously, the resulting bone collagen isotope values are consistent with a high trophic position, similar to previously generated values of *A. simus* in Canada and Alaska^18,21,34^ (Table 1). We used the R package simmr^35,36^ to estimate the proportional contributions of terrestrial and marine food sources to this individual’s diet. We incorporated stable isotope values from the published literature that best match the plausible spatial and temporal resources available to *A. simus* and thus avoid potential issues associated with baseline shifts^37^ and Suess effects^38^. We used values of Late Pleistocene (~ 15–30,000 years old) terrestrial megafauna from Rancho La Brea including bison (*Bison antiquus*), camel (*Camelops hesternus*), horse (*Equus occidentalis*) ground sloth (*Paramylodon harlani*), and mastodon (*Mammut americanum*)^39,40^ (Supplemental Table 3). We combined bison and camels into a single ruminant category as they did not have significantly different means for both carbon and nitrogen. As a proxy for marine food consumption, we used pinnipeds from archaeological sites on San Miguel Island (Supplemental Table 3)^41^. Across three simmr models with differing resource inputs, we estimated that pinnipeds accounted for only ~ 16–22% of diet contributions, with the majority of diet contributions instead coming from terrestrial animal protein such as bison and camels. (Supplemental Table 4, Supplemental Fig. 2). While no analogous stable isotope datasets are available to infer the diet of CCI pygmy mammoths, dental microwear analyses suggest that they were browsers consuming twigs and leaves^42^, consistent with pollen-based reconstructions of Late Pleistocene forests on the CCI^43^. Therefore, a hypothetical insular population of *A. simus* would have had access to terrestrial resources of largely C3 signatures, lessening (but not entirely removing) the potential to conflate C4 prey resources with a marine signature.

### Radiocarbon calibration and extinction modeling

**Figure 4:**
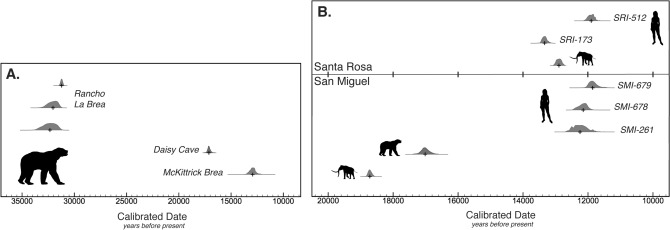
(**A**) *Arctodus* radiocarbon dates from across California on bone collagen (Supplemental Table 6). (**B**) Radiocarbon dates from early human occupation sites on the Channel Islands and late-surviving mammoths in comparison with *A. simus*. Human-associated localities are noted by their site names. SRI-512: OS-75147, charcoal; SRI-173: CAMS-17215, bone; SMI-679: OS-80171, marine shell; SMI-678: OS-80129, marine shell^44,81^. The pygymy mammoth date from Santa Rosa was directly dated bone collagen (CAMS-71697), whereas the date from San Miguel is stratigraphically associated charcoal (WW-10415)^55^. All dates have been appropriately calibrated according to the material dated (IntCal13 vs. MarineCal13 with local marine reservoir correction)^82^. Note that there are several available dates for the site SRI-173^81^, and we conservatively present the oldest available to highlight the temporal gap between humans and the *A. simus* specimen. Animal symbols courtesy of Phylopic. Figures produced using OxCal^45^ and Adobe Illustrator.

Stable isotope mixing models indicated a small dietary contribution of marine resources to overall carbon values. Therefore, we used a mixed curve IntCal13 80%/MarineCal13 20% with a local reservoir correction^44^ of 225 ± 35, using OxCal^45^ v. 4.3. With these conditions, the calibrated age is a mean of 17,009 ± 135 cal ybp (median 17,016 cal ybp; 95.4% range of 16,713–17,296 cal ybp); a traditional terrestrial only calibration with IntCal13 would be similar with a 95.4% range of 16,955–17,460 cal ybp (Supplemental Table 5). This date is consistent with the known temporal range of *A. simus* from California (Fig. 4A). In all calibration options, the CCI *A. simus* material predates Terminal Pleistocene Paleoindian localities on Santarosae (Fig. 4B). However, when conservatively considering the potential evidence for an ephemeral human occupation on Santarosae at ~ 18,000 cal ybp^9^, humans would have been at the site roughly a millennium before the bear died, though additional evidence is required to substantiate this scenario.

We employed the Gaussian-resampled inverse-weighted McInerny (GRIWM) method to estimate the probable extinction timing of *A. simus*, an approach which accounts for uncertainty in the fossilization and detection process^46^. Using all quality-controlled, direct radiocarbon dates available for the species from across the North American range^17^ (n = 34) and only those post-Last Glacial Maximum (n = 12) (Supplemental Table 6), we found that *A. simus* may have persisted until ~ 12–13,000 years before present based on the GRIWM method (Supplemental Table 7). However, when including only radiocarbon dates from California (n = 5), we found the method poorly predicts likely persistence by extrapolating a last occurrence in the Late Holocene of ~ 3–4,000 years before present, likely reflecting the small sample size and gaps in sampling for this region. While our new date allows us to meet the minimum suggested threshold of five dates to run GRIWM, it is clear that additional dates are required to more accurately resolve extinction timing at the regional level of California.

## Discussion

The diet of *A. simus* has been debated for decades, as modern bears are highly flexible in how they use geographically variable resources, and we lack comparable datasets across *A. simus*’ full range. Collagen stable isotopes from Beringia^18^ (δ^13^C − 18.27 ± 0.4‰, δ^15^N 8.5 ± 1.02‰) and British Columbia^34^ (δ^13^C − 18.9‰, δ^15^N 10.6‰) are suggestive of a high trophic position with terrestrial meat consumption, especially caribou^20^. However, definitive calculation of trophic position, particularly in the case of omnivorous mammals that readily consume meat and plant material, must rely on future analyses of compound specific stable isotopes of individual amino acids and larger sample sizes^47,48^. Brown bears, *U. arctos*, tend to consume more animal protein at northern latitudes^49^, and coastal populations in Alaska today exhibit stable isotope values with more positive carbon values than that of the CCI *A. simus*, reflecting more marine protein consumption (δ^13^C − 15.46 ± 2.04‰, δ^15^N 10.79 ± 4.15‰; Suess corrected^18^).

The large sample size of *A. simus* cranial material from California (disproportionately from Rancho La Brea) has yielded dental-based inferences of diet that do not align with the meat-heavy diets of northern populations. The presence of dental caries suggests a high carbohydrate intake for Los Angeles-area populations^22^, and dental microwear attributes conflict with bone consumption associated with scavenging^23^. Carbon isotopes from inland California *A. simus* enamel indicate a signal of C3 vegetation, but there is no corroborating nitrogen value to distinguish animal protein consumption^50^. This CCI specimen thus represents the first isotopic inference of *A. simus* diet south of Canada, with a δ^15^N value (13.2‰) and mixing model results (Supplemental Table 4) that indicate consumption of animal protein, supporting previous findings from northern areas. However, we stress that this single individual may not fully represent the dietary breadth of the species in California.

**Figure 5:**
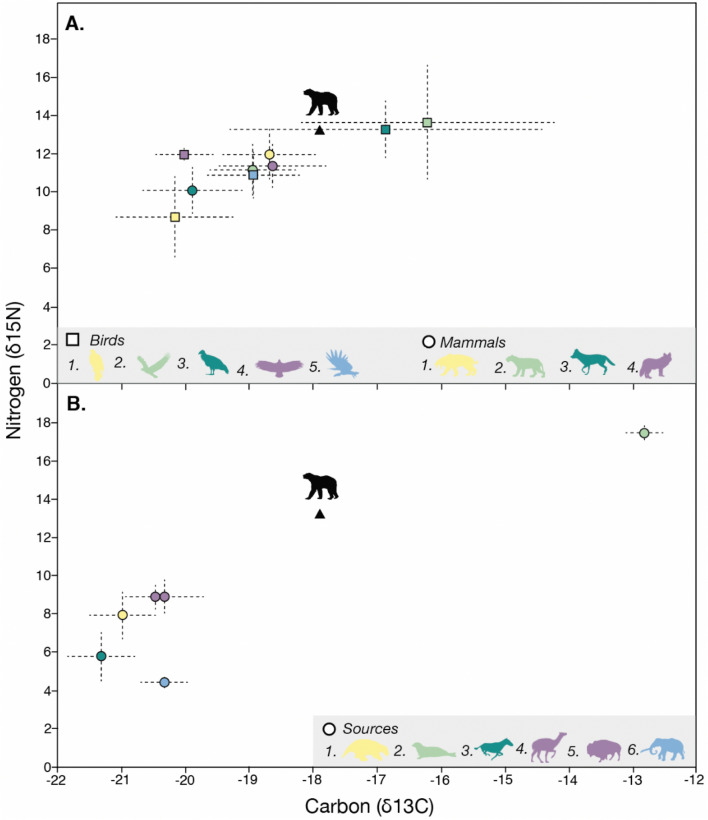
Stable carbon (x-axis) and nitrogen (y-axis) isotope values for varius species discussed in this study. (**A**) Birds^59^: 1. Golden eagle, *Aguila chryseatos* (n = 2), 2. Bald eagle, *Haliaeetus leucocephalus* (n = 8), 3. California condor, *Gymnogyps californianus* (n = 9), 4. Black vulure, *Coragyps occidentalis* (n = 8), 5. Merriam’s teratorn, *Teratornis merriami* (n = 6). Mammals^39^: 1. Saber-tooth cat, *Smilodon fatalis* (n = 23), 2. American lion, *Panthera atrox* (n = 8), 3. Coyote, *Canis latrans* (n = 9), 4. Dire wolf, *Canis dirus* (n = 25). (**B**) Sources^39–41^: 1. Ground sloth, *Paramylodon harlani* (n = 10), 2. Pinnipeds, *Callorhinus ursinus* & *Phoca vitulina* (n = 16) 3. Western horse, *Equus occidentalis* (n = 19), 4. Yesterday’s camel, *Camelops hesternus* (n = 12), 5. Antique bison, *Bison antiquus* (n = 31) (4 & 5 are grouped as “ruminants”), 6. American mastodon, *Mammut americanum* (n = 7). Animal symbols courtesy of Phylopic. Figures were produced using the statistical software R v3.6.2 (including simmr^35,36^) and Adobe Illustrator.

The rich record of Rancho La Brea allows us to contextualize this species within its contemporaneous Late Pleistocene ecosystem (Fig. 5). The CCI individual exhibits a larger nitrogen value than all other co-occurring mammalian and avian predators, and most closely resembles the California condor (*Gymnogyps californianus*), which in the Late Pleistocene consumed a diet of 20–30% marine protein^40^. The *A. simus* δ^13^C value (− 17.9‰), coupled with mixing model results (Supplemental Fig. 1), indicate similar partial marine resource use for this individual, which may have sustained short-faced bears in the presence of a much denser megafaunal carnivore guild. Compared with CCI predators in the Holocene, *A. simus* shows more marine resource use than the island fox^12^ (*Urocyon littoralis*, δ^13^C − 18.34 ± 1.10‰, δ^15^N 9.51 ± 1.95‰), but less than humans^11,51^ (δ^13^C − 14.09 ± 0.76‰, δ^15^N 16.10 ± 1.68‰) and bald eagles (δ^13^C − 13.88 ± 1.55‰, δ^15^N 16.79 ± 1.84‰)^52^. Though we are limited to a single individual and more study is required to understand population-wide dietary trends, we tentatively suggest that *A. simus* was an opportunistic scavenger of both marine and terrestrial carcasses across a wide home range, aligning with reconstructions based on Beringian specimens^18^. This increased resolution into the basic natural history of *A. simus*, particularly its plausible use of a coastal environment, permits us to more robustly address our biogeographic hypotheses.

First, we consider the pre-mortem hypotheses: that (1) there was an established population of *A. simus* on Santarosae and (2) the bone represents a single natural dispersal event, in which an individual swam to Santarosae and died there. The specimen’s presence in a cave setting is consistent with the denning behavior of extant bears, and several *A. simus* individuals have been found deceased in a denning position (e.g., Big Bear Cave in Missouri^53^). Santarosae was recurrently colonized by Columbian mammoths with early overwater immigration events of MIS 6 (~ 150 ka) or 8 (~ 250 Ka)^5^; there is no evidence for the presence of *A. simus* at this time on the CCI, though they were present in mainland California at this time (Fig. 1) and during the Irvingtonian^50^. The last sea level minimum occurred ~ 20,000 years ago, with levels > 100 m lower than today, exposing extensive and now submerged portions of coastline and Santarosae reaching its largest land area of 2,147 square km^6^. Sea levels rose after this, and with a calibrated range of 16,713–17,296 cal ybp, our *A. simus* died at a time when the shortest distance between CCI and the mainland was ~ 8 km (today’s shortest distance is ~ 19 km^6^). While extant bears are strong swimmers, *A. simus* is only known from one other insular locality, Vancouver Island, and it is rare there despite a largely continental fauna, represented by an isolated ulna in a glacial gravel pit^34^ and another individual with multiple associated skeletal elements in a cave^54^.

Both taphonomic conditions and biogeographic dispersal filters argue against a pre-mortem presence of *A. simus* on Santarosae. With the occurrence of just a single skeletal element, there is no evidence for a standing population of *A. simus*, or any other bears, on the island, especially considering the extensive paleontological work on the island and the abundant fossil record of pygmy mammoths, which are known from multiple skeletal elements and individuals documented for nearly 400 open air localities^55^. Even in the case of an individual dispersal event, the presence of a single isolated metapodial in a cave, with no other skeletal elements in a well-known site is striking—unless an isolated bone or paw was scavenged from a dead bear that was already on the island and carried to the site by a bald eagle or condor, both of which are known to transport animal bones^56^. More than 100 *A. simus* localities from across North America have revealed a distinct taphonomic pattern^57^: individuals from open-air sites have a select number of elements recovered, whereas cave sites have relatively complete skeletons, likely representing individuals that died during denning^53^.

This leaves two post-mortem transport hypotheses to explain the presence of *A. simus* in Daisy Cave: either (3) humans or (4) another agent carried the metacarpal to the site. Multiple Indigenous cultures in California interacted with *Ursus* bears, using claws and bones for ornamental and ritual objects, but these are rare and usually represented by a single element or individual, such as the clearly modified femora from Late Holocene cultural contexts on San Miguel, Santa Rosa, and San Nicolas sites^58^. The Daisy Cave metapodial shows no sign of human modification and no recognizable human-made artifacts were present. While there was likely temporal overlap between humans and *A. simus* on the mainland and evidence for long-distance trade networks linking the CCI and mainland peoples as early as ~ 11,700 years ago^44^, this significantly post-dates the age of our *A. simus* specimen (Table 1). Santarosae has one of the earliest directly dated Paleoindian skeletons in North America, Arlington Springs Man (CA-SRI-173, Fig. 1), dated to ~ 13,000 cal B.P, overlapping with the apparent age of Stratum I at Daisy Cave. Therefore, it is theoretically conceivable (if unlikely) that the *A. simus* bone was collected by humans on the islands or the adjacent mainland after its death, curated as an heirloom ritual item for a millennium or more, then deposited at Daisy Cave ~ 13,000 years ago. The good preservation of both collagen and DNA and lack of permineralization of the specimen, however, argues against any extended exposure prior to deposition in Daisy Cave.

The placement of a roughly 17,000-year-old *A. simus* bone within Daisy Cave’s well-defined strata I and J, dated between ~ 13,000 and 14,000 cal ybp, is difficult to explain. In the absence of firm evidence for an anthropogenic translocation, it seems more likely that another vector transported the specimen, post-mortem, to the CCI. Southern California had a rich Late Pleistocene avian community, including now extirpated and extinct birds of prey such as Merriam’s teratorn (*Teratornis merriami*), golden eagles (*Aguila chrysaetos*), bald eagles (*Haliaeetus leucocephalus*), California condors (*Gymnogyps californianus*), and black vultures (*Coragyps occidentalis*). Stable isotope data from Rancho La Brea reveal suggest that several of these species utilized marine resources^52,59^ (Fig. 5A), supporting a potential foraging connection between mainland and island ecosystems. If, as our stable isotope results suggest, *A. simus* was opportunistically foraging in coastal areas, then it is plausible its carcass would have been within the range of an avian scavenger from the CCI. Raptor roosts, especially those presumed to be bald eagles and barn owls, are common fossil deposits in the CCI^10,60,61^, and raptors are the primary taphonomic agent for small mammals within Daisy Cave^13^. A scenario in which a raptor incorporated the *A. simus* metapodial from the mainland into its nest within Daisy Cave is plausible, thereby capturing physical evidence of a rare ecological interaction. This scenario still must explain the several millennia gap in the age of the *A. simus* bone and Strata I and J at Daisy Cave. The bone could have initially been deposited on a higher rocky shelf located inside the rockshelter^8^ , then displaced several millennia later by subsequent animal (or human) activity; this mechanism has also been used to explain the presence of extinct mouse bones in Late Holocene strata at nearby Cave of the Chimneys (CA-SMI-603)^13^.

## Conclusion

The histories of island ecosystems worldwide bear the imprint of geologic, climatic, and human legacies, spanning temporal scales ranging from millennia to decades. This complex layering highlights the pivotal role of past anthropogenic activities in shaping modern patterns of diversity and divergence as revealed by the archaeological record. Using a suite of interdisciplinary methods and a context-dependent hypothesis testing framework, we identified an unexpected mainland species from an insular context outside of its known biogeographic range and systematically evaluated potential mechanisms for its occurrence on the CCI. Our work highlights the need to avoid assumptions regarding the presence of specimens in mixed archaeological and paleontological deposits and to carefully consider site, specimen, and regional chronologies when determining potential agents of dispersal. A human-mediated translocation cannot be ruled out for the Daisy Cave *A. simus* specimen and might have been an obvious hypothesis given the specimen’s presence in a well-known archaeological deposit. Yet, our approach ultimately revealed another biotic interaction—avian scavenging—as an alternative, and potentially more likely explanation. We also emphasize the importance of archaeological datasets and the continually evolving toolkits of ancient biomolecules in generating insights into enigmatic species histories, with opportunities to enrich fundamental knowledge of island ecology, evolution, and conservation. Our isotopic data, while limited to a single individual, provides a foundation for testing future hypotheses of the late survival of *A. simus* in California, as its opportunistic marine foraging and flexible diet may have facilitated persistence in the face of environmental change similar to that of the California Condor^59^, contributing to a larger dialogue surrounding the extinction of Late Quaternary megafauna.

## Methods

### Excavation methods

Daisy Cave (CA-SMI-261) is an important archaeological site with some of the earliest evidence of human occupation in the Americas, as documented by a series of excavations^8^ conducted from the 1960s and 1990s. The *A. simus* bone was recovered in 1993 during excavations led by Erlandson. Excavation was performed by hand trowel and finer methods (e.g., straws, dental picks, and small brushes), following natural stratigraphic layers, and using a combination of 1/8 and 1/16-inch mesh to screen sediments in the field. Because of a dearth of burrowing rodents and relatively dry, sheltered conditions, the deposits at Daisy Cave are exceptionally well preserved, including preservation of perishable woven sea grass artifacts. A robust radiocarbon chronology demonstrates that Daisy Cave was occupied by humans between ~ 12,000–800 cal BP (Strata A-G), and possibly as early as 18,000 years ago, with the cave interior used for human burials^8,62^ (Supplemental Fig. 1). The *A. simus* bone was recovered in Stratum I from Unit D6 (southwestern ½ × 1) located along the rock shelter drip line. After excavation, all materials were transported to the University of Oregon for analysis, where they were rinsed in tap water and subsequently stored in a climate-controlled laboratory.

### Radiocarbon dating and stable isotope analysis

The metapodial was processed at the Penn State Human Paleoecology and Isotope Geochemistry Laboratory. We began with an initial sample weight of 250 mg and a crude gelatin yield of 11.9%, which is approximately half the expected yield for fresh bone (20–25% by weight). The gelatin was ultrafiltered to a > 30 kDa fraction with a yield of 6.9%. An aliquot of 2.20 mg of collagen was subsequently combusted and sent to UCIAMS as CO_2_ for graphitization and AMS analysis. 0.73 mg of collagen was sent to Yale for analyses of %C, %N, δ^13^C and δ^15^N.

We used the R package simmr^35,36^ to estimate the proportional contributions of terrestrial and marine food sources to the value of isotopes. We collected stable isotope values from the published literature that best match the spatial and temporal resources available to *A. simus* and thus avoid potential issues associated with baseline shifts^37^ and Suess effects^38^. Given the body size of *A. simus* we did not include smaller terrestrial or marine prey items that would likely not be target species (e.g., rodents, small fish). By including a range of primary consumers, we cover the range of potential consumers of local C3 and C4 plants in our model. No species-specific trophic enrichment factor (TEF) exists for *A, simus* or its closest living relative, *Tremarctos ornatus*. Therefore, we applied a collagen-collagen TEF developed for Late Pleistocene carnivores^63^ of ∆^13^C 1.1 ± 0.2‰ and ∆^15^ N 3.8 ± 1.1‰.

### Collagen fingerprinting (ZooMS)

Approximately 50 mg of the CCI specimen was processed for ZooMS collagen fingerprinting^64^. In brief this involved removal of collagen through decalcification with 0.6 M hydrochloric acid overnight and ultrafiltration into 50 mM ammonium bicarbonate using 10 kDa molecular weight cut-off filters with two exchanges. The protein component was then digested with 0.4 µg sequencing grade trypsin at 37 °C overnight. The digests were then acidified to 0.1% trifluoroacetic acid (TFA) and then ziptipped with C18 (Varian OMIX) pipette tips for peptide purification, eluted in 50% acetonitrile (ACN) in 0.1% TFA and dried to completion by centrifugal evaporation. Samples were then rehydrated with 10 µL 0.1% TFA and 1 µL co-crystalised with an equal amount of 10 mg/mL alpha-cyano hydroxycinnamic acid in 50% ACN/0.1% TFA and allowed to dry. Peptide mass fingerprints were then acquired using a Bruker Ultraflex II Matrix Assisted Laser Desorption Ionization Time of Flight Mass Spectrometer over the *m/z* range 700–3,700 and compared with reference spectra for mammals^29,65^.

### Ancient DNA

DNA was extracted in the Laboratories of Molecular Anthropology and Microbiome Research’s dedicated ancient DNA laboratory. In brief, the exterior of the bone was decontaminated with 1.5% bleach, one minute of UV crosslinking on each side of the bone, and the outer surface was removed by a Dremel. Both the sample (138.8 mg of bone) and a negative extraction control were processed^66^. Following a partial uracil-DNA-glycosylase treatment^67^, Illumina sequencing libraries were constructed^68^ (see Supplementary Materials). To enrich for mitochondrial DNA, dual indexed libraries were captured with in-solution custom design RNA baits, following the manufacturer’s protocol (Arbor Biosciences, Ann Arbor, USA) with minor changes. Libraries were sequenced on the Illumina MiSeq platform (2 × 150 bp) in the Consolidated Core Laboratory at the University of Oklahoma.

Raw reads were quality-filtered using AdapterRemoval2 (-minlength 30, -trimqualities, -minquality 20, -trimns, -collapse) and merged reads were mapped with BWA v. 0.7.17 with the seed disabled and the edit distance set to 0.01 to several bear and human reference mitochondrial genomes (Supplementary Table 8). Duplicates and low-quality mapped reads (less than q37) were removed with samtools v. 1.5. Bam files were visually inspected and consensus sequences were generated for the alignment to *A. simus* in Geneious v. 10.0.8. Almost all reads mapping to other bear references overlapped with those mapping to *A. simus* (Supplementary Fig. 3). MapDamage2 was used to investigate fragment misincorporation frequency^69^, however due to the high clonality and low endogenous DNA content, it was not possible to clearly authenticate with fragment misincorporation profiles (Supplementary Fig. 4) nor use coverage cutoffs for consensus sequences. The consensus sequence was aligned to six bear mitogenomes using MAFFT^70^ v 7.45 as implemented in Geneious v. 10.0.8. To place the consensus sequence in a phylogenetic framework, we inferred a phylogeny with the Maximum Likelihood method and Hasegawa-Kishino-Yano model^71^ in MEGA X^72,73^. This model was chosen for the dataset using Akaike's Information Criterion for all models in MEGA X on a partial deletion (90%) alignment with a total of 5,435 positions and the giant panda, *Ailuropoda melanoleuca*, as an outgroup^32^. A discrete Gamma distribution was used to model evolutionary rate differences among sites. The tree with the highest log likelihood is shown and the percentage of trees from 1,000 bootstrap replicates in which the associated taxa clustered together is shown next to the branches^74^. Sequences are deposited in the Sequence Read Archive.

## Supplementary information

Supplementary Information.

Supplementary Datasets.

Supplementary Data: DNA alignment

## Data Availability

All relevant data, scans, sequence alignments, supplemental figures, tables, and methods have been uploaded as electronic supplemental material. Sequence data can be downloaded from the Sequence Read Archive:  http://www.ncbi.nlm.nih.gov/bioproject/657837.
