# Supplementary material for: Biogeographic problem-solving reveals the Late Pleistocene translocation of a short-faced bear to the California Channel Islands
Source: Sci Rep. 2020 Sep 16;10:15172. doi: 10.1038/s41598-020-71572-z (PMC7494929; doi:10.1038/s41598-020-71572-z)
Supplement: Supplementary file 1 — Supplementary Information. [file 41598_2020_71572_MOESM1_ESM.docx]

**SUPPLEMENTAL MATERIAL
Biogeographic problem-solving reveals the Late Pleistocene translocation of a short-faced bear to the California Channel Islands**

Alexis M. Mychajliw^1,2,3*^, Torben C. Rick^4^, Nihan D. Dagtas^1,2^, Jon M. Erlandson^5,6^, Brendan J. Culleton^7^, Douglas J. Kennett^8^, Michael Buckley^9^, Courtney A. Hofman^1,2,4^

1. Department of Anthropology, University of Oklahoma, Norman OK
2. Laboratories of Molecular Anthropology and Microbiome Research, University of Oklahoma, Norman OK
3. La Brea Tar Pits and Museum, Los Angeles CA
4. Department of Anthropology, National Museum of Natural History, Smithsonian Institution, Washington DC
5. Museum of Natural and Cultural History, University of Oregon, Eugene OR
6. Department of Anthropology, University of Oregon, Eugene OR
7. Institutes of Energy and the Environment, The Pennsylvania State University, University Park, PA 16802, USA
8. Department of Anthropology, University of California, Santa Barbara, Santa Barbara CA
9. School of Natural Sciences, Manchester Institute of Biotechnology, University of Manchester, M1 7DN, UK

*Correspondence to: mych0000@ou.edu

**Supplemental Figures**

**
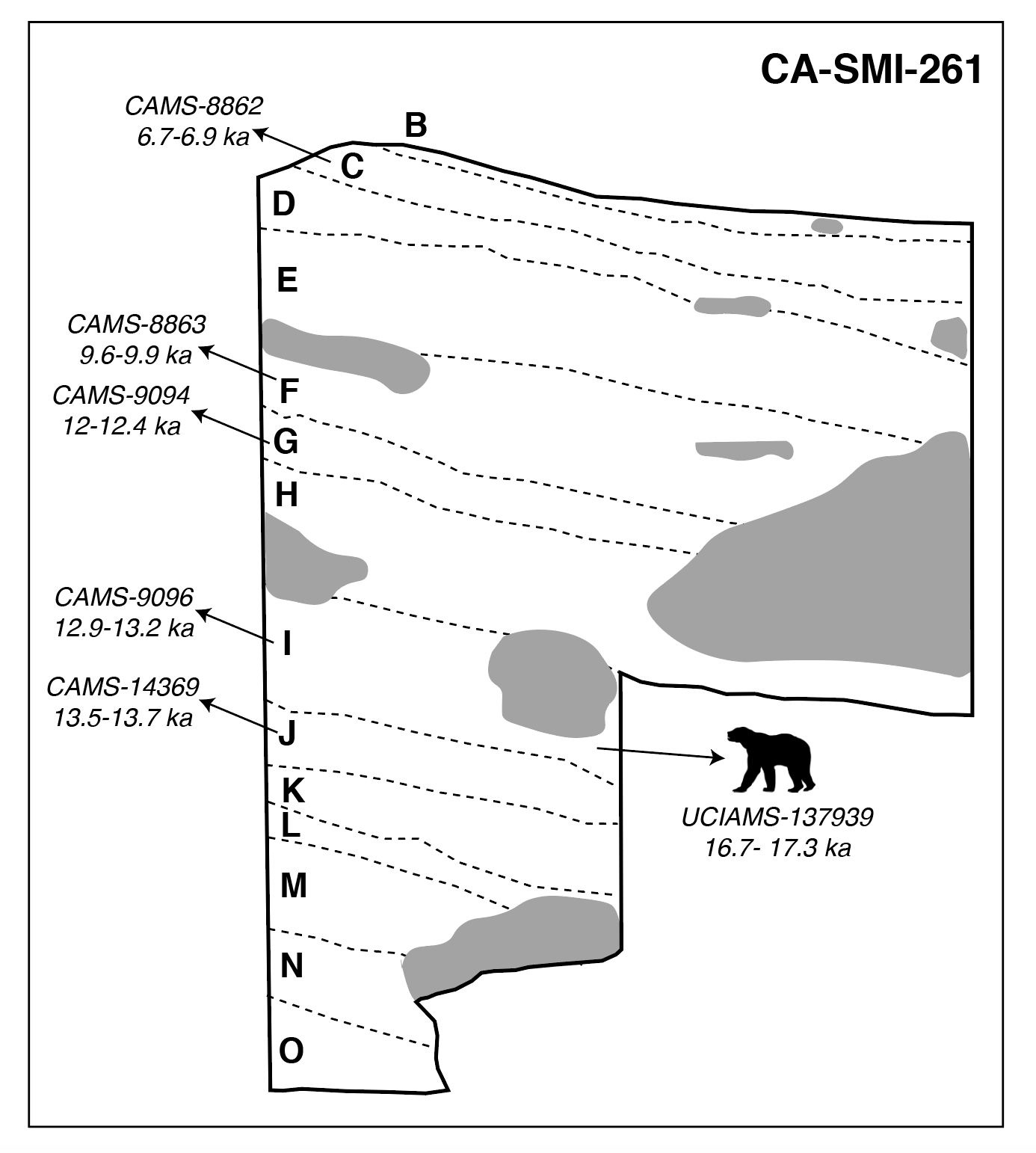
**

**Supplemental Figure 1.** Simplified stratigraphic profile from Daisy Cave, San Miguel Island, California Channel Islands (CA-SMI-261) as adapted from Erlandson et al (1996). Dates are calibrated calendar year ranges from Erlandson et al (1996). We refer interested readers to Erlandson et al (1996) for details on stratigraphic profiles and excavation methods. Letters refer to stratigraphic levels, with specific dates called out from Erlandson et al (1996). Gray shapes indicate roof spall. *Arctodus simus* image courtesy of PhyloPic. Figure was produced in Adobe Illustrator.

Erlandson J. et al. An archaeological and paleontological chronology for Daisy Cave (CA-SMI-261), San Miguel Island, California. *Radiocarbon* **38**, 355-373 (1996).


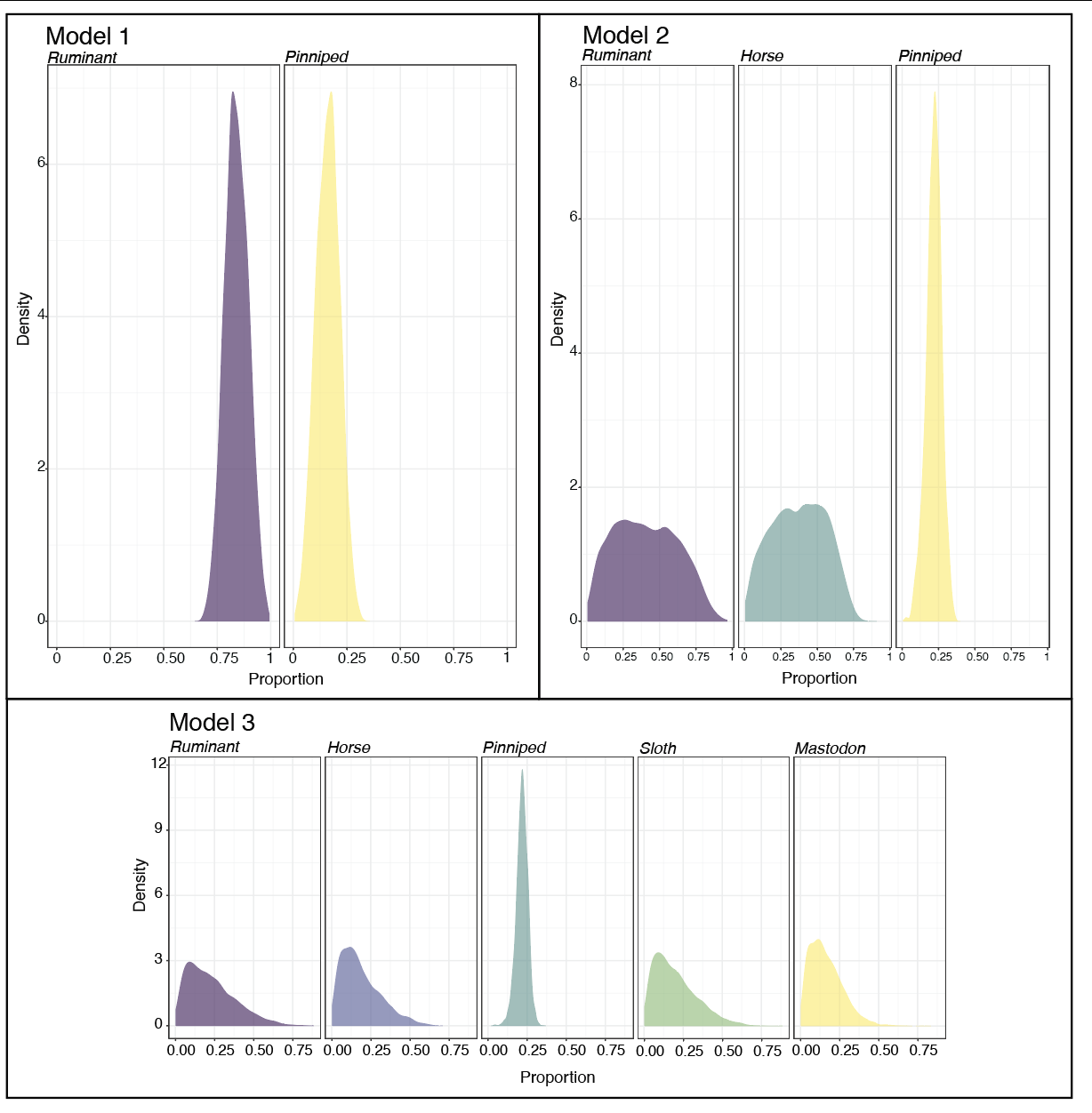


**Supplemental Figure 2.** Proportional dietary contributions of different food sources based on simmr models. Figures were produced using the statistical software R. Models correspond to those in Supplemental Table 4.


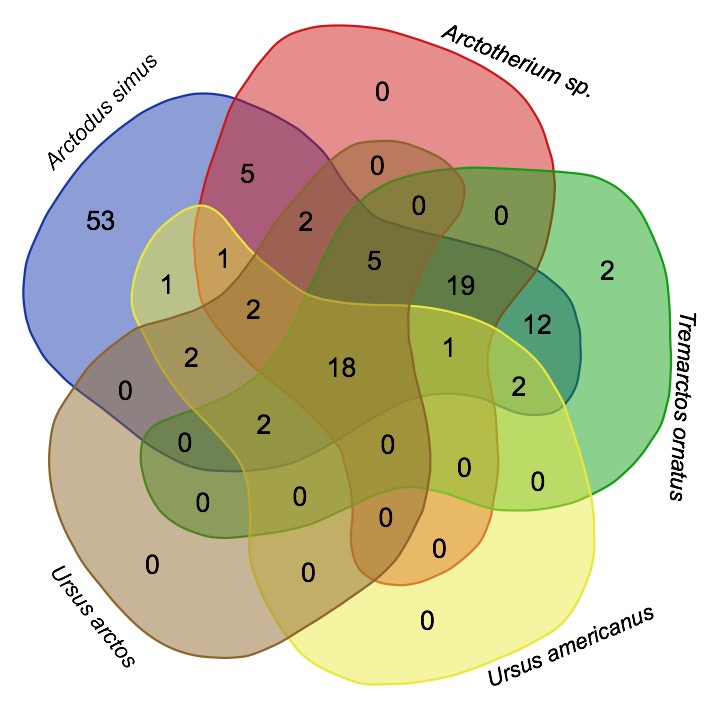


**Supplementary Figure 3**. Venn Diagram of unique reads mapping to each reference mitochondrial genome. Nearly all unique reads (except two to *Tremarctos ornatus)* mapped to *Arctodus simus* in addition to mapping to other reference sequences. Therefore, the consensus sequence of this bone mapped to *Arctodus simus* likely captures all genetic variation present. Venn Diagram made with tools at <http://bioinformatics.psb.ugent.be/webtools/Venn/>

**Supplementary Figure 4.** Fragment misincorporation plot for Channel Islands Bear mapped to *Arctodus simus*.

**Supplemental Tables**

**Supplemental Table 1**. Hypotheses tested in our study and putative lines of evidence that would be congruent with a given scenario. Hypotheses were developed based on geology (Pleistocene sea level fluctuations), biogeography (patterns of other mainland and island species), dispersal biology (swimming potential, range, and diet), and archaeology (timing of human presence).

|  | **Hypothesis** | **Abundance** | **Skeletal Elements** | **Specimen Age** |
| --- | --- | --- | --- | --- |
| **Pre-mortem** | Standing population | Multiple individuals across a variety of deposits, similar to pygmy mammoths | Multiple skeletal elements would be represented in a cave deposit | Contemporaneous with pygmy mammoths (Late Pleistocene) |
|  | Single dispersal event | A single individual | Multiple skeletal elements would be represented in a cave deposit | Contemporaneous with pygmy mammoths (Late Pleistocene) |
| **Post-mortem** | Human transport | Rare, restricted to deposits with clear human activity (e.g., middens) | An isolated element, potentially modified | After initial human arrival, ~12,000 years or more recent |
|  | Avian transport | Rare, restricted to deposits with evidence of raptor roosts | An isolated element of suitable size | Any time when *A. simus* was extant |

**Supplemental Table 2**. Comparison of metapodial measurements from the literature. MC = metacarpal; MT = metatarsal; GL = greatest length; DPE = Diameter of proximal end; LWS = least width of shaft; GBD = greatest breadth of distal end. Measurements in mm. ^1^This study. ^2^Merriam & Stock 1925, ^3^Kurten 1967, ^4^Schubert & Kaufmann 2003, ^5^Emslie & Czaplewski 1985, ^6^Burns & Young 2011, ^7^Pasenko 2018, ^8^Harrington et al 2014, ^9^Kuijper et al 2016.

| **Element** | **Locality** | **Specimen** | **GL** | **DPE** | **LWS** | **GBD** | **Species** |
| --- | --- | --- | --- | --- | --- | --- | --- |
| MC1 | Daisy Cave | UO 14-6778^1^ | 71.03 | 24.33 | 11.12 | 17.62 | *Arctodus simus* |
| MC1 | Potter Creek Cave | UCMP 3040^2^ | 72.6 | 24.2 | 10.8 | 19.5 | *Arctodus simus* |
| MC1 | Potter Creek Cave | UCMP 8291^3^ | 75 |  | 11.4 | 20.7 | *Arctodus simus* |
| MC1 | Big Bear Cave | ISM 496850^4^ | 75.1 |  |  | 19.2 | *Arctodus simus* |
| MC1 | Rancho La Brea | UCMP 12768^2^ | 75.7 | 23.5 | 10.6 | 19 | *Arctodus simus* |
| MC1 | Frankstown Cave | CM 11019^3^ | 80 | 25.1 | 10.7 | 19.6 | *Arctodus simus* |
| MC1 | Potter Creek Cave | UCMP 3116^3^ | 80 |  | 12.3 | 21 | *Arctodus simus* |
| MC1 | Rancho La Brea | LACM Z93^2^ | 81.5 | 24.8 | 11.6 | 21.2 | *Arctodus simus* |
| MC1 | Rancho La Brea | UCMP 17754^2^ | 86.8 | 29.8 | 13.9 | 25.7 | *Arctodus simus* |
| MC2 | Potter Creek Cave | UCMP 3040^2^ | 96.8 | 30 | 13.4 | 23.3 | *Arctodus simus* |
| MC2 | Big Bear Cave | ISM 496850^4^ | 103 |  |  | 24.4 | *Arctodus simus* |
| MC2 | Frankstown Cave | CM 11019^3^ | 106 | 33.7 | 15.2 | 25 | *Arctodus simus* |
| MC2 | Rancho La Brea | UCMP 14816^2^ | 114.3 | 36.5 | 18.6 | 29.4 | *Arctodus simus* |
| MC2 | Frankstown Cave | CM11020^3^ | 126 | 40.8 | 18.8 | 29.8 | *Arctodus simus* |
| MC3 | Potter Creek Cave | UCMP 3040^2^ | 104 | 31.6 | 14 | 26.5 | *Arctodus simus* |
| MC3 | Potter Creek Cave | UCMP 3042^3^ | 106 |  | 14.8 | 26 | *Arctodus simus* |
| MC3 | Rancho La Brea | UCMP 21004^2^ | 107.6 | 33.3 | 14.6 | 24.8 | *Arctodus simus* |
| MC3 | Big Bear Cave | ISM 496850^4^ | 109.5 |  |  | 24.4 | *Arctodus simus* |
| MC3 | Frankstown Cave | CM 11019^3^ | 112 | 33.7 | 15.2 | 25 | *Arctodus simus* |
| MC3 | Potter Creek Cave | UCMP 5019^3^ | 113 |  | 15.5 | 26.6 | *Arctodus simus* |
| MC3 | Rancho La Brea | UCMP 17754^2^ | 126.7 | 39.3 | 18.7 | 29.9 | *Arctodus simus* |
| MC3 | Hay Springs | FAM 25536^3^ | 134 | 44 | 22.4 | 35.1 | *Arctodus simus* |
| MC3 | Friesenhahn Cave | UTTMM 933^3^ |  | 30.3 | 14.2 |  | *Arctodus simus* |
| MC3 | Labor-of-Love Cave | LACM 122434^5^ |  | 31.1 | 13.5 |  | *Arctodus simus* |
| MC4 | Potter Creek Cave | UCMP 3040^2^ | 106.3 | 31 | 15.8 | 25.7 | *Arctodus simus* |
| MC4 | Potter Creek Cave | UCMP 3041^3^ | 107 |  | 15.5 |  | *Arctodus simus* |
| MC4 | Big Bear Cave | ISM 496850^4^ | 110.6 |  |  | 26.1 | *Arctodus simus* |
| MC4 | Frankstown Cave | CM 11019^3^ | 113 | 33.2 | 15.2 |  | *Arctodus simus* |
| MC4 | Rancho La Brea | LACM Z124^2^ | 123 | 36.9 | 18.6 | 29.7 | *Arctodus simus* |
| MC4 | Rancho La Brea | UCMP 17754^2^ | 130.5 | 41.7 | 23 | 34 | *Arctodus simus* |
| MC5 | Potter Creek Cave | UCMP 3040^2^ | 104 | 32.3 | 16.1 | 25.5 | *Arctodus simus* |
| MC5 | Potter Creek Cave | UCMP 3041^3^ | 105 | 32.3 |  |  | *Arctodus simus* |
| MC5 | Big Bear Cave | ISM 496850^4^ | 111.1 |  |  | 27.3 | *Arctodus simus* |
| MC5 | Rancho La Brea | LACM Z117^2^ | 128.3 | 38.1 | 20.8 | 32.6 | *Arctodus simus* |
| MC5 | Rancho La Brea | UCMP 17754^2^ | 130.2 | 43.5 | 21 | 34 | *Arctodus simus* |
| MT1 | Rock Creek | AMNH 12392^3^ | 72 | 28.8 | 13.8 | 21.5 | *Arctodus simus* |
| MT1 | Rancho La Brea | LACM Z101^2^ | 73.2 | 27.7 | 10.8 | 18.3 | *Arctodus simus* |
| MT1 | Big Bear Cave | ISM 496850^4^ | 74.6 |  |  | 18 | *Arctodus simus* |
| MT1 | Rancho La Brea | LACM Z102^2^ | 75.2 | 32 | 12 | 21.3 | *Arctodus simus* |
| MT1 | Hay Springs | FAM 25536^3^ | 81 | 30 | 16.4 | 21.6 | *Arctodus simus* |
| MT2 | Rock Creek | AMNH 12392^3^ | 89 | 38.7 | 15.2 | 21.8 | *Arctodus simus* |
| MT2 | Big Bear Cave | ISM 496850^4^ | 94.7 |  |  | 23.6 | *Arctodus simus* |
| MT2 | Rancho La Brea | LACM Z40^2^ | 101.3 | 36.8 | 16.3 | 24.8 | *Arctodus simus* |
| MT3 | Big Bear Cave | ISM 496850^4^ | 103.4 |  |  | 23.3 | *Arctodus simus* |
| MT3 | Rancho La Brea | LACM Z99^2^ | 106.3 | 37.8 | 16 | 23.2 | *Arctodus simus* |
| MT3 | Yukon | NMC-36237^6^ | 115.4 | 36.4 | 21 |  | *Arctodus simus* |
| MT3 | Edmonton | P93.8.41^6^ | 115.5 | 41.1 | 20.1 | 26.3 | *Arctodus simus* |
| MT3 | Edmonton | P89.13.91^6^ | 118.5 | 44.8 | 15.9 | 25.3 | *Arctodus simus* |
| MT3 | Rancho La Brea | LACM Z90^2^ | 121.2 | 41.8 | 20.8 | 30.5 | *Arctodus simus* |
| MT3 | Hay Springs | FAM 25536^3^ | 124 | 75 | 22.7 |  | *Arctodus simus* |
| MT4 | Frankstown Cave | CM 11019^3^ | 106 | 34.7 | 17.7 |  | *Arctodus simus* |
| MT4 | Rock Creek | AMNH 12392^3^ | 108 | 35.8 | 17.4 |  | *Arctodus simus* |
| MT4 | Big Bear Cave | ISM 496850^4^ | 114.1 |  |  | 24.9 | *Arctodus simus* |
| MT4 | Rancho La Brea | LACM Z99^2^ | 115.7 | 38 | 17.4 | 25.2 | *Arctodus simus* |
| MT4 | Rancho La Brea | LACM Z88^2^ |  | 40.6 | 19.8 |  | *Arctodus simus* |
| MT5 | Rancho La Brea | UCMP 24251^2^ | 110 | 39.8 | 13.8 | 24.3 | *Arctodus simus* |
| MT5 | Rock Creek | AMNH 12392^3^ | 113 | 38.7 | 16.9 |  | *Arctodus simus* |
| MT5 | Big Bear Cave | ISM 496850^4^ | 113.1 |  |  | 25.7 | *Arctodus simus* |
| MT5 | RLB | LACM Z99^2^ | 115.8 | 37 | 15.6 | 24.5 | *Arctodus simus* |
| MT5 | RLB | LACM Z85^2^ | 129 |  | 19.8 | 30.8 | *Arctodus simus* |
| MT5 | Frankstown Cave | CM 11019^3^ |  | 36 |  |  | *Arctodus simus* |
| MC1 | Arizona | SHM 17844^7^ | 52 |  |  |  | *Ursus americanus* |
| MC2 | Arizona | SHM 17844^7^ | 63 |  |  |  | *Ursus americanus* |
| MC3 | Arizona | SHM 17844^7^ | 67 |  |  |  | *Ursus americanus* |
| MC4 | Arizona | SHM 17844^7^ | 69 |  |  |  | *Ursus americanus* |
| MC5 | Arizona | SHM 17844^7^ | 71 |  |  |  | *Ursus americanus* |
| MT3 | Canada | CMN 41055^8^ | 68.4 | 15 | 10.5 |  | *Ursus americanus* |
| MT3 | Canada | CMN 1583^8^ | 66.5 | 14.8 | 9.6 |  | *Ursus americanus* |
| MT3 | Canada | CMN 75389^8^ | 67.1 | 14.1 | 9 |  | *Ursus americanus* |
| MT3 | Canada | CMN 34109^8^ | 64.4 | 11.5 | 8.8 |  | *Ursus americanus* |
| MT3 | Canada | CMN 5009^8^ | 61.1 | 12.2 | 8.3 |  | *Ursus americanus* |
| MT3 | Canada | CMN Z-162^8^ | 57.6 | 10.6 | 8.7 |  | *Ursus americanus* |
| MC1 | Netherlands | Unknown^9^ | 66.5 | 20.1 |  |  | *Ursus arctos* |
| MC2 | Netherlands | Unknown^9^ | 75.6 | 15.8 |  |  | *Ursus arctos* |
| MC3 | Netherlands | Unknown^9^ | 75.3 | 15.1 |  |  | *Ursus arctos* |
| MC4 | Netherlands | Unknown^9^ | 78.3 | 15.5 |  |  | *Ursus arctos* |
| MC5 | Netherlands | Unknown^9^ | 78.9 | 20 |  |  | *Ursus arctos* |
| MT3 | Canada | MPEP 82.1^8^ | 72.6 | 16.1 | 10.8 |  | *Ursus arctos* |
| MT3 | Canada | CMN 41056^8^ | 79 | 18.6 | 13.5 |  | *Ursus arctos* |
| MT3 | Canada | CMN 2772^8^ | 82.4 | 19 | 12.6 |  | *Ursus arctos* |
| MT3 | Canada | CMN 31187^8^ | 84 | 19.9 | 13.0 |  | *Ursus arctos* |

1. This study.
2. Merriam, J. C. & Stock, C. Relationship and structure of the short-faced bear *Arctotherium*, from the Pleistocene of California. *Carnegie Inst. Washington* **437**, 1-35 (1925).
3. Kurten, B. Pleistocene bears of North America, Part 2. Genus *Arctodus*, short-faced bears. *Acta Zool. Fennica* **117**, 1-60 (1967).
4. Schubert, B. W. & Kaufmann, J. E. A partial short-faced bear skeleton from an Ozark cave with comments on the paleobiology of the species. *Journal of Cave and Karst Studies* **65**, 101-110 (2003).
5. Emslie, S. D. & Czaplewski, N. J. A new record of the giant short-faced bear, *Arctodus simus*, from western North America with a reevaluation of its paleobiology. *Natural History Museum of Los Angeles County Contributions in Science* **371**, 1-12 (1985).
6. Burns, J. A. & Young, R. R. Pleistocene mammals of the Edmonton area, Alberta. Part I. The carnivores. *Canadian Journal of Earth Sciences* **31**, 393-400 (2011).
7. Pasenko, M. R. A partially articulated black bear (*Ursus americanus*) from Late Quaternary deposits in the Tonto National Forest, Central Arizona. *J of the Arizona-Nevada Academy of Science* **47**, 28-35 (2018).
8. Harington, C. R., Cournoyer, M., Chartier, M., Fulton, T. L. & Shapiro B. Brown bear (*Ursus arctos*) (9880±35 BP) from late-glacial Champlain Sea deposits at Saint-Nicolas, Quebec, Canada, and the dispersal history of brown bears. *Canadian Journal of Earth Sciences* **51**, 527-535 (2014).
9. Kuijper, W. J., Verheijen, I. K. A., Ramcharan, A., van der Plicht, H. & van Kolfschoten, T. One of the last wild brown bears (Ursus arctos) in the Netherlands (Noordwijk). *Lutra* **59**, 49-64 (2016).

**Supplemental Table 3.** Potential dietary sources as used in SIMMR. All values represent bone collagen. ^1^Coltrain et al 2004 from Rancho La Brea; ^2^Chamberlain et al 2005 from Rancho La Brea; ^3^Newsome et al 2007 from San Miguel Island.

| **Species** | **Category** | **N** | **δ^13^C Mean ± sd** | **δ^15^N Mean ± sd** |
| --- | --- | --- | --- | --- |
| *Camelops hesternus*^1^ | Terrestrial | 12 | -20.48 ± 0.34 | 8.89 ± 0.66 |
| *Equus occidentalis*^1,2^ | Terrestrial | 19 | -21.33 ± 0.54 | 5.78 ± 1.30 |
| *Paramylodon harlani*^1^ | Terrestrial | 10 | -20.99 ± 0.52 | 7.93 ± 1.26 |
| *Bison antiquus*^1,2^ | Terrestrial | 31 | -20.33 ± 0.62 | 8.92 ± 0.90 |
| *Mammut americanum*^1^ | Terrestrial | 7 | -20.33 ± 0.37 | 4.41 ± 0.31 |
| *Callorhinus ursinus, Phoca vitulina*^3^ | Marine | 16 | -12.8 ± 0.3 | 17.5 ± 0.4 |

1. Coltrain, J. B. et al. Rancho La Brea stable isotope biogeochemistry and its implications for the paleoecology of late Pleistocene, coastal southern California. *Palaeogeography, Palaeoclimatology, Palaeoecology* **205**, 199-219 (2004).
2. Chamberlain, C. P. et al. Pleistocene to recent dietary shifts in California condors. *Proceedings of the National Academy of Sciences* **102**, 16707-16711 (2005).
3. Newsome, S. D. et al. The shifting baseline of northern fur seal ecology in the northeast Pacific Ocean. *Proceedings of the National Academy of Sciences* **104**, 9709-9714 (2007).

**Supplemental Table 4.** SIMMR model outputs of proportional diet contribution estimates for different food sources. The ruminant category includes both bison and camel. See Supplemental Table 3 for isotopic values of food sources.

| **Source** | **Model 1** | **Model 2** | **Model 3** |
| --- | --- | --- | --- |
| Ruminant | 0.84 ± 0.055 | 0.408 ± 0.215 | 0.229 ± 0.152 |
| Pinniped | 0.16 ± 0.055 | 0.218 ± 0.053 | 0.217 ± 0.037 |
| Horse |  | 0.374 ± 0.182 | 0.187 ± 0.125 |
| Sloth |  |  | 0.202 ± 0.136 |
| Mastodon |  |  | 0.165 ± 0.105 |

**Supplemental Table 5.** Differing calibration schemes employed, including the use of terrestrial and marine curves and inclusion of marine reservoir correction. Calibrations were carried out using OxCal v. 4.3 (Bronk Ramsey 2009). *Delta_R = 225 ± 35 (Erlandson et al 2011).

| **Calibration** | **Mean** | **σ** | **Median** | **95.4% Range** |
| --- | --- | --- | --- | --- |
| IntCal13 100% | 17200 | 129 | 17198 | 16955-17460 |
| MarineCal13 20% | 17080 | 130 | 17079 | 16820-17361 |
| MarineCal13 20%, Delta_R* | 17170 | 135 | 17016 | 16713-17296 |
| MarineCal13 100%, Delta_R* | 16260 | 132 | 16254 | 16004-16539 |

**Supplemental Table 6.** Compilation of all direct radiocarbon dates for *Arctodus simus* across pre and post Last Glacial Maximum (~21-18,000 years before present) periods.

*Dates combined pre-calibration in Oxcal v. 4.3 using the “combine” function, as they are multiple dates on the same individual. Chi square test to confirm combination is statistically appropriate.

| **Locality** | **Lab #** | **Element** | **^14^C age** | **Median cal ybp** | **Reference** |
| --- | --- | --- | --- | --- | --- |
| Friesenhahn Cave, Texas | NZA-28895 | Dentine (m3) | 10814 ± 55 | 12718 | Schubert 2010 |
| Bonner Springs, Kansas | NZA-28889 | Lumbar vertebra | 10921 ± 50 | 12776 | Schubert 2010 |
| Huntington, Utah | AA-6974  NZA-28855 | Maxilla | 10953 ± 36 | 12788 | Madsen 2000;  Schubert 2010 |
| McKittrick, California | CAMS-138657 | Ulna | 11040 ± 310 | 12936 | Fox-Dobbs et al 2014 |
| Sheridan Cave, Ohio^*^ | CAMS-12837,  NZA-28856,  CAMS-33968,  CAMS-12839,  CAMS-12845 | Scapholunars, Astragali | 11556 ± 25 | 13397 | Tankersley et al 1997; Tankersley et al 1999; Schubert 2010 |
| Rochester, Indiana | GX-12483 | Rib | 11500 ± 520 | 13473 | Richards & Turnbull 1995 |
| Bonner Springs, Kansas | NZA-28890 | Femur | 11688 ± 50 | 13513 | Schubert 2010 |
| Pellucidar, Vancouver Island* | UCIAMS-41049,  OxA-24005,  UCIAMS-41048 | Palatine, M2, humerus | 11700 ± 20 | 13517 | Steffen & Fulton 2018 |
| Lake Bonneville, Utah | Unknown | Femur | 12650 ± 70 | 15037 | Nelson & Madsen 1983 |
| San Miguel Island, California | UCIAMS- 137939 | Metapodial | 14130 ± 70 | 17016 | This paper |
| Saltville Valley, Virginia | NZA-21830 | Dentine (m2) | 14853 ± 55 | 18058 | Schubert & Wallace 2009 |
| Perkins Cave, Missouri | CAMS-77882 | Dentine | 16910 ± 50 | 20399 | Schubert 2004 |
| *Last Glacial Maximum* | | | | | |
| La Sena, Nebraska | NZA-28896 | Dentine (I3) | 19487 ± 95 | 23477 | Schubert 2010 |
| Ophir Creek, Canada | Beta-79852 | Unknown | 20210 ± 110 | 24280 | Matheus 1997 |
| Fairbanks, Alaska | AA-17511 | Unknown | 20524 ± 180 | 24727 | Matheus 1997 |
| Eldorado Creek, Canada | Wk20235 | Right calcaneum | 22417 ± 452 | 26713 | Krause et al 2008 |
| Vancouver Island, Canada | UCIAMS-56480 | Right ulna | 22750 ± 140 | 27106 | Steffen & Harrington 2010 |
| Hunker Creek, Canada | TO-3707 | Unknown | 24850 ± 150 | 28884 | Matheus 1997 |
| Alaska | Wk20236 | Humerus | 25264 ± 650 | 29450 | Bray 2011 |
| Fairbanks, Alaska | AA-17512 | Unknown | 25496 ± 224 | 29632 | Matheus 1997 |
| Gold Run Creek, Canada | TO-2696 | Unknown | 26040 ± 270 | 30284 | Matheus 1997 |
| Hester Creek, Canada | OxA-9259 | Ulna | 26720 ± 270 | 30899 | Bray 2011 |
| Quartz Creek, Canada | ANUA-38615 | Unknown | 26940 ± 570 | 31029 | Bray 2011 |
| Ikpikpuk River, Alaska | TO-2539 | Humerus | 27160 ± 280 | 31142 | Churcher et al 1993 |
| Rancho La Brea,  California | CAMS-119957 | Left humerus | 27330 ± 140 | 31217 | Fuller et al 2015 |
| Upper Cleary Creek, Alaska | AA-17513 | Unknown | 27511 ± 279 | 31355 | Matheus 1997 |
| Rancho La Brea,  California | CAMS-119965 | Right metatarsal | 28130 ± 330 | 32040 | Fuller et al 2015 |
| Rancho La Brea,  California | CAMS-119956 | VI cervical vertebra | 28350 ± 470 | 32335 | Fuller et al 2015 |
| Lower Hunker Creek, Yukon | I-11037 | Humerus | 29600 ± 1200 | 33744 | Harrington 1980 |
| Gittin Down Mt. Cave, Oklahoma | NZA-27734 | Dentine (m2) | 34063 ± 460 | 38550 | Schubert 2010 |
| Island Ford Cave, Virginia | NZA-23693 | Dentine (m1) | 34080 ± 480 | 38569 | Schubert 2010 |
| Birch Creek, Alaska | AA-17515 | Unknown | 34974 ± 652 | 39576 | Matheus 1997 |
| Fairbanks, Alaska | AA-17514 | Unknown | 39565 ± 1126 | 43536 | Matheus 1997 |
| Sixty Mile, Canada | TO-2699 | Unknown | 44240 ± 930 | 47621 | Matheus 1997 |

Bray, S. C. 2011. Mitochondrial DNA analysis of the evolution and genetic diversity of ancient and extinct bears. PhD Thesis, University of Adelaide (2011).

Churcher, C. S., Morgan, A. V. & Carter, L. D. *Arctodus simus* from the Alaskan arctic slope *Canadian Journal of Earth Sciences* **30**, 1007-1013 (1993).

Fox-Dobbs, K., Leonard J. A., & Koch, P. L. Pleistocene megafauna from eastern Beringia: paleoecological and paleoenvironmental interpretations of stable carbon and nitrogen isotope and radiocarbon records. *Palaeogeography, Palaeoclimatology, Palaeoecology* **261**, 30-46 (2008).

Fuller, B. T., Harris, J. M., Farrell, A. B., Takeuchi, G. & Southon, J. R. Sample preparation for radiocarbon dating and isotopic analysis of bone from Rancho La Brea. *Natural History Museum of Los Angeles County Science Series* **42**, 151-167 (2015).

Harington, C. R. Radiocarbon dates on some Quaternary mammals and artifacts from northern North America. *Arctic* **33**, 815-832 (1980).

Krause, J. et al. Mitochondrial genomes reveal an explosive radiation of extinct and extant bears near the Miocene-Pliocene boundary*. BMC Evolutionary Biology* **8**, 220 (2008).

Madsen, D. B. A high-elevation Allerød-Younger Dryas megafauna from the west-central Rocky Mountains. In: Madsen. D.B., Metcalf, M.D. (Eds.), Intermountain Archaeology. University of Utah Anthropological Papers No. 122, Salt Lake City, pp. 100–113 (2000).

Matheus, P. Paleoecology and ecomorphology of the giant short-faced bear in Eastern Beringia. PhD Thesis, Univesity of Alaska, Fairbanks (1997).

Nelson, M. E. & Madsen, J. H. A giant short-faced bear (*Arctodus simus*) from the Pleistocene of Utah. *Transactions of the Kansas Academy of* Science **86**, 1-9 (1983).

Richards, R. L. & Turnbull, W. D. Giant short-faced bear (*Arctodus simus yukonensis*) remains from Fulton County, northern Indiana. *Fieldiana: Geology* **30**, 1-34 (1995).

Schubert, B. W. A full-glacial short-faced bear (*Arctodus simus*) from Perkins Cave, Missouri. *Current Research in the Pleistocene* **21**, 115-116 (2004).

Schubert, B. W. Late Quaternary chronology and extinction of North American giant short-faced bears (*Arctodus*). *Quaternary International* **217**, 188-194 (2010).

Schubert, B. W. & Wallace, S. C. Late Pleistocene giant short-faced bears, mammoths, and large carcass scavenging in the Saltville Valley of Virginia, USA. *Boreas* **38**, 482-492 (2009).

Steffen, M. L. & Fulton, T. L. On the association of giant short-faced bear (*Arctodus simus*) and brown bear (*Ursus arctos*) in late Pleistocene North America. *Geobios* **51**, 61-74 (2018).

Steffen, M. L. & Harington, C. R. Giant short-faced bear (*Arctodus simus*) from late Wisconsinan deposits at Cowichan Head, Vancouver Island, British Columbia. *Canadian Journal of Earth Sciences* **47**, 1029-1036 (2010).

Tankersley, K. B. Sheriden: a stratified Pleistocene-Holocene cave site in the Great Lakes region of North America. *British Archaeological Research International Series* **800**, 67-75 (1999).

Tankersley, K. B., Ford, K. M., McDonald, H. G., Genheimer, R. A. & Hendricks, R. Late-Pleistocene archaeology of Sheriden Cave, Wyandot County, Ohio. *Current Research in the Pleistocene* **14**, 81-83 (1997).

**Supplemental Table 7.** Gaussian-resampled inverse-weighted McInerny (GRIWM) results across multiple subsets of radiocarbon dates, in calibrated years before present.

| **Subset** | **n** | **2.5% upper CI** | **Median** | **97.5% lower CI** |
| --- | --- | --- | --- | --- |
| California | 5 | 1753 | 3413 | 5103 |
| Post-LGM | 12 | 11383 | 12505 | 12735 |
| All Direct Dates | 34 | 11155 | 12432 | 12720 |

**Supplemental Table 8.** Sequence statistics. ^1^After trimmed and merged,

^2^Mapped reads /Analysis-ready reads, ^3^Mapped reads/ Unique Q37 mapped reads

| **Reference** | **GenBank Accession #** | **Sample** | **Total Reads** | **Analysis-ready reads^1^** | **Proportion kept reads^1^** | **Mapped reads** | **Proportion of reads mapped^2^** | **Q37 mapped reads** |
| --- | --- | --- | --- | --- | --- | --- | --- | --- |
| *Arctodus*  *simus* | FM177762.1 | CI-Bear | 2,011,568 | 1,866,293 | 0.9277 | 1901 | 0.001 | 1901 |
| *Arctodus*  *simus* | FM177762.1 | CI-Bear-ExtNeg | 151,501 | 125,282 | 0.8269 | 0 | 0 | 0 |
| *Tremarctos*  *ornatus* | EF196665.1 | CI-Bear | 2,011,568 | 1,866,293 | 0.9277 | 1347 | 0.0007 | 1067 |
| *Tremarctos*  *ornatus* | EF196665.1 | CI-Bear-ExtNeg | 151,501 | 125,282 | 0.8269 | 0 | 0 | 0 |
| *Arctotherium*  *sp.* | NC_030174.1 | CI-Bear | 2,011,568 | 1,866,293 | 0.9277 | 1185 | 0.0006 | 762 |
| *Arctotherium*  *sp.* | NC_030174.1 | CI-Bear-ExtNeg | 151,501 | 125,282 | 0.8269 | 0 | 0 | 0 |
| *Ursus*  *arctos* | AF303110.1 | CI-Bear | 2,011,568 | 1,866,293 | 0.9277 | 775 | 0.0004 | 634 |
| *Ursus*  *arctos* | AF303110.1 | CI-Bear-ExtNeg | 151,501 | 125,282 | 0.8269 | 0 | 0 | 0 |
| *Homo*  *sapien* | NC_012920.1 | CI-Bear | 2,011,568 | 1,866,293 | 0.9277 | 178 | 0 | 159 |

| **Reference** | **Unique Q37 mapped reads** | **Average length of mapped reads** | **Rescaled or Not** | **Mean coverage** | **Std dev of mean coverage** | **% reference covered >= 1X** | **Cluster factor^3^** |
| --- | --- | --- | --- | --- | --- | --- | --- |
| *Arctodus*  *simus* | 125 | 58.728 | Yes | 0.4382X | 0.6998X | 33.29% | 15.208 |
| *Arctodus*  *simus* | 0 | No unique mapped reads | BAM file was empty, truncated, or not generated | N/A | N/A | N/A | N/A |
| *Tremarctos*  *ornatus* | 61 | 55.1311 | Yes | 0.2006X | 0.5183X | 15.57% | 22.0819 |
| *Tremarctos*  *ornatus* | 0 | No unique mapped reads | BAM file was empty, truncated, or not generated | N/A | N/A | N/A | N/A |
| *Arctotherium*  *sp.* | 53 | 54.1321 | Yes | 0.1735X | 0.4803X | 13.63% | 22.36 |
| *Arctotherium*  *sp.* | 0 | No unique mapped reads | BAM file was empty, truncated, or not generated | N/A | N/A | N/A | N/A |
| *Ursus*  *arctos* | 31 | 50.4839 | Yes | 0.092X | 0.3675X | 6.83% | 25 |
| *Ursus*  *arctos* | 0 | No unique mapped reads | BAM file was empty, truncated, or not generated | N/A | N/A | N/A | N/A |
| *Homo*  *sapien* | 8 | 47.375 | No | 0.0228X | 0.1829X | 1.71% | 22.25 |

**Supplemental Methods**

*Mitogenome Reconstruction*

Sample preparation and DNA extraction

For decontamination purposes the sample was gently wiped once with a tissue damped with 1.5 % NaClO and once with dH_2_O, exposed crosslinking with UV light for 1 min on each side. The outer surface then was scraped, a piece was cut out by using a Dremel diamond blade and pulverized using a sterilized hammer. The total amount of bone powder going into the extraction was 138.8 mg. The extraction batch also included a negative control.

The following is a modified version of the extraction method used in Dabney et al. 2013. Extraction started with an EDTA wash step, where 1 ml of 0.5M EDTA was added to the bone powder and powder was resuspended by vortexing. Then the tubes were put on a nutator for 15 min. This step further helps eliminate contamination originating from the environment. After incubation, tubes were centrifuged at 13k rpm for 3 min, the supernatant was discarded and fresh 1 ml 0.5M EDTA was added to the pellet. Pellet was resuspended by briefly vortexing the tube and the tubes were left on a rotator for overnight incubation at room temperature. Next day, after a brief spin to move any liquid away from the cap, 100 ul of Qiagen Proteinase K (Cat. no. 19133) was added to the tubes and tubes were left on the rotator for another round of incubation for 3 days. After incubation the tubes were centrifuged at 13k rpm for 3 min and the supernatant was transferred to a new tube (labelled EDTA-A) and kept at 4^o^C for later use. One ml fresh 0.5 M EDTA and 50 ul Proteinase K was added to the pellet and pellet was resuspended by vortexing. The tubes were incubated at room temperature on a rotator for another 5 days, until most of the pellet is dissolved exhibiting buoyancy.

Before transferring the supernatant, Zymo reservoirs (Cat. no. C103125) were UV treated and reservoir-Qiagen MinElute column (Cat. no. 28006) apparatus was manually assembled using force. Then 13 ml Qiagen PB buffer was added into the assembled apparatus and the sample tubes were centrifuged for 2 min at 13k rpm. Supernatant was then transferred to the apparatus containing PB, by slowly pipetting to mix the contents. Supernatant kept in EDTA-A tubes was also transferred on top of that and mixed well by pipetting. The apparatus was centrifuged for 4 min at 1500 g, then rotated 90^o^ and centrifuged for another 2 min. The flow-through was discarded and the filtered column was washed twice using 650 ul Qiagen PE buffer, centrifuging at 10k rpm for 1 min in between washes. Tubes were dry spun for an additional minute at 13k rpm to eliminate any remaining EtOH. Columns were transferred to new 1.5 ml tubes and 30 ul Qiagen EB buffer was added onto the filter and tubes were incubated at room temperature for 5 min, then centrifuged at 13k rpm for 1 min. Addition of 30 ul EB buffer and the steps after that were performed again until a DNA extract of 60 ul was collected. The extracts were quantified using Qubit 3.0 fluorometer (Thermo Fisher Scientific, Waltham USA) with dsDNA HS assay kit (Cat. no. Q32854) following manufacturer’s protocol. DNA concentration of the bear extract was 0.77 ng/ul, while the extraction negative was too low to be detected.

Single tube DNA library preparation

Preparation of adapters: Following Meyer & Kircher (2010), a 10X oligo hybridization buffer was prepared with final concentrations of 500mM NaCl, 10mM Tris-Cl (pH 8), and 1mM EDTA (pH 8). The P5 and P7 adapters (using the modified IS3_BEDC3 adapter from Caroe et al. (2018) were mixed in separate PCR tubes and incubated in a thermocycler at 95^o^C for 10 sec, followed by a ramp from 95 to 12^o^C at a rate of 0.1^o^C/sec. The two reactions were then combined to obtain an adapter mix.

Preparation of libraries: Illumina sequencing libraries were built following Caroe et al. (2018) with minor modifications. Library building started with a partial UDG treatment step as in Rohland et al. (2015). The final concentration of reagents used in this step was as follows; 1X NEB Buffer2, 0.1 uM dNTP mix, 0.1 mg/ml BSA, 1.2 mM ATP, 0.072 U/ul USER enzyme, mixed with 9.7 ul of DNA extract in a final volume of 14 ul. The reaction was then incubated for 30 min at 37^o^C and for 1 min at 12^o^C. A microliter of UGI enzyme was then added to each library individually. Tubes were flicked and quickly spun, then incubated at 37^o^C for 30 min and at 12^o^C for 1 min. For the end repair step, final concentrations of 1X T4 DNA ligase reaction buffer, 0.5 uM dNTP mix, and 0. 12 ul End Repair enzyme mix were mixed and distributed to the tubes containing 15 ul of UDG-treated DNA. Tubes were flicked and quickly spun before they were incubated for 30 min at 20^o^C and for another 30 min at 65^o^C. For adapter ligation, first an aliquot of 1 ul 10 uM adapter mix was added to each tube separately and mixed by pipetting. Then 3 ul of a master mix containing 0.4 ul of Quick ligation buffer, 2.5 ul of PEG 4000 (50%), and 0.1 ul of Quick T4 ligase was added to the tubes and mixed well. The tubes were incubated at 20^o^C for 30 min. For the adapter fill-in step, a master mix was prepared using 3 ul of Isothermal amplification buffer, 0.3 ul of 25 mM dNTP mix, 6.2 ul molecular grade H_2_O, 0.5 ul Bst 2.0 Warmstart polymerase and 10 ul aliquots were added to the adapter ligated libraries. Tubes were incubated at 65^o^C for 20 min, and then at 80^o^C for 20 min. After fill-in step, a 1.5X bead clean-up using Sera-mag Speedbeads (Fisher, Cat. no. 09-981-123) was performed. 46.7 ul beads were added to each reaction and tubes were incubated at room temperature for 5 min. They were then placed on a magnetic particle collector for 5 min and the supernatant was discarded. Beads were washed on the magnet twice with 100 ul 80% ethanol, let dry for 3-4 min to remove remaining EtOH. 25 ul Qiagen EB buffer was added to the dry beads and beads were resuspended. After incubation at room temperature for 5 min, tubes were again put on the magnetic particle collector and clean libraries were eluted and collected in clean tubes.

Quantification of libraries and double indexing

Before libraries were indexed for Illumina sequencing, a quantification must be performed to determine the number of PCR cycles needed. For that purpose, we performed a qPCR quantification on LightCycler 96 (Roche, Switzerland) using a master mix prepared with the final concentrations of 1X KAPA HiFi+Uracil mix, 0.1 mg/ml BSA, 0.3 uM forward primer, 0.3 uM reverse primer, and 8 uM SYTO 9 Green. Aliquots of 24 ul of master mix were distributed to PCR tubes and 1 ul from each library was added to the tubes. The qPCR profile was as follows; initial denaturation at 94^o^C for 5 min, followed by 45 cycles of denaturation at 98^o^C for 20 sec, annealing at 50^o^C for 15 sec, elongation at 72^o^C for 30 sec. Depending on the plateau phase for each sample, number of PCR cycles were determined. They were 16, 25 and 23 cycles for the sample, extraction negative and library negative, respectively.

For double indexing a master mix containing 6 ul H_2_O, 12.5 ul KAPA HiFi HotStart Uracil+ mix and 1 ul 2.5 mg/ml BSA was prepared. After distributing 19.5 ul of the mix to individual tubes, 4 ul of library template was added. As the last component, 0.75 ul of forward and reverse indexing primers were each added to the tubes so that each sample has a unique pair of indices. All indexing reactions were performed in triplicates. The PCR profile contained an initial denaturation step at 95^o^C for 5 min, followed by a denaturation segment at 98^o^C for 20 sec, annealing segment at 60^o^C for 15 sec and elongation segment at 72^o^C for 30 sec (different number of cycles for each sample) and a final elongation at 72^o^C for a minute. The PCR products were checked on 2% agarose gels before pooling the replicates. Pooled replicates were cleaned up using Sera-mag Speedbeads as explained in the “Preparation of libraries” section. Indexed and cleaned libraries were then quantified using Qubit 3.0 fluorometer with dsDNA HS assay kit (Cat. no. Q32854) following manufacturer’s protocol. DNA concentrations were 38.2, 40.2 and 28.8 ng/ul for the bear, extraction negative and library negative libraries, respectively. Later, these samples were run on the Fragment Analyzer (Agilent, Santa Clara USA) using the NGS kit (DNF-474) to assess the fragment length distribution and DNA concentration in the desired range.

MyBaits In-Solution Capture for mitogenome enrichment

The bait used to enrich this bone sample for bear DNA was designed to target a diverse group of mammals, fish, amphibians, reptiles, and birds living on the California Channel Islands. This probe set was designed from 225 input sequences (105 from CO1 gene, 108 from Cytochrome b gene and 12 complete mitogenomes). It also included a complete *Ursus americanus* mitogenome (NC_003426.1) in light of the discovery of this bone. Probes were generated according to MyBaits standards as 80mer probes with 4 x tiling density yielding 18,052 unique probes.

Hybridization capture of mtDNA fragments was performed following MyBaits v3.02 manual with slight modifications. We used half the amount of probes stated in the original manual. We also followed a touchdown strategy for the hybridization step, with a temperature range of 65 – 55^o^C for 47 hours. Enriched libraries were qPCR quantified on LightCycler 96 using a master mix composed of 7 ul nuclease free H_2_O, 12.5 ul 2X KAPA Hifi HS Ready mix, 1 ul 2.5 mg/ml BSA, 0.75 ul IS5 and IS6 primers each (as in Meyer & Kircher, 2010), and 2 ul SYTO 9 Green. A microliter of enriched library was mixed with 24 ul of the master mix, and amplified using the following PCR profile: preincubation at 95^o^C for 2 min, a 45 cycle 3-step amplification at 98^o^C for 20 sec, 60^o^C for 15 sec, 72^o^C for 30 sec, cooling at 72^o^C for 1 min. After quantification, all samples were amplified for 25 cycles to increase the number of enriched molecules for efficient sequencing. PCR master mix was prepared using 5 ul nuclease free H_2_O, 25 ul 2X KAPA HiFi HS Ready mix, 2.5 ul of 10 uM IS5 and IS6 each, and 15 ul of enriched library on-beads. Amplified enriched libraries were then taken off the beads on a magnetic particle collector, and then subjected to 1.8X bead clean-up following the protocol described above.

Quantification, pooling and preparation for sequencing

Quantification was done using Qubit 3.0 fluorometer with dsDNA HS assay kit and two microliters of each sample were diluted to approximately 1 ng/ul to be run on the Fragment Analyzer (Agilent, Santa Clara USA). Samples were then pooled in equimolar concentrations using the concentration values within the 150-500 bp range after running the Fragment Analyzer NGS kit (DNF-474). The sequencing pool was dried down to 30 ul on Savant DNA 120 Speedvac (Thermo Fisher Scientific, Waltham USA) and mixed with 10 ul of the marker supplied with the 2% agarose gel cassette (CDF2010) for Pippin Prep (Sage Science, USA). Adapter dimers and other impurities were eliminated and only the fragments within the 150-500 bp range were eluted on the Pippin Prep run. The cleaned pool was later quantified using the following protocol on LightCycler 96: initial denaturation at 95^o^C for 5 min, and 35 cycles of denaturation at 95^o^C for 30 sec and acquisition at 60^o^C for 45 sec. This run was set up using 12 ul of the KAPA SYBR FAST qPCR Master Mix + Primer Premix (Roche, Cat. no. KK4824) and 4 ul PCR grade water for 4 ul of each sample or standard (KAPA Library Quantification Standards, Cat. no. KK4824). The pool was diluted, denatured and prepared for sequencing following the Illumina MiSeq System Denature and Dilute Libraries Guide.

Sequencing

The pool was sequenced on a single lane of Illumina MiSeq flowcell using a v2 paired-end 2x150bp kit. Sequencing was carried out at the Consolidated Core Laboratory of the University of Oklahoma. Demultiplexing was performed on Illumina’s BaseSpace and subsequent analyses were performed on the servers of the Laboratories of Molecular Anthropology and Microbiome Research.

References

Caroe, C., Gopalakrishnan, S., Vinner, L., Mak, S. S. T., Sinding, M. H. S., Samaniego, J. A., Wales, N., Sicheritz-Ponten, M. & Gilbert, T. P. Single-tube library preparation for degraded DNA. *Methods in Ecology and Evolution* **9**, 410-419 (2018).

Meyer, M. & Kircher, M. Illumina sequencing library preparation for highly multiplexed target capture and sequencing. Cold Spring Harbor Protocols, 6: pdb.prot5448 (2010).

Rohland, N., Harney, E., Mallick, S., Nordenfelt, S. & Reich, D. Partial uracil-DNA-glycosylase treatment for screening of ancient DNA. *Philos Trans R Soc Lond B Biol Sci*. **370**, 20130624 (2015).
