# Supplementary figures and images for: Biogeographic problem-solving reveals the Late Pleistocene translocation of a short-faced bear to the California Channel Islands
Source: Sci Rep. 2020 Sep 16;10:15172. doi: 10.1038/s41598-020-71572-z (PMC7494929; doi:10.1038/s41598-020-71572-z)

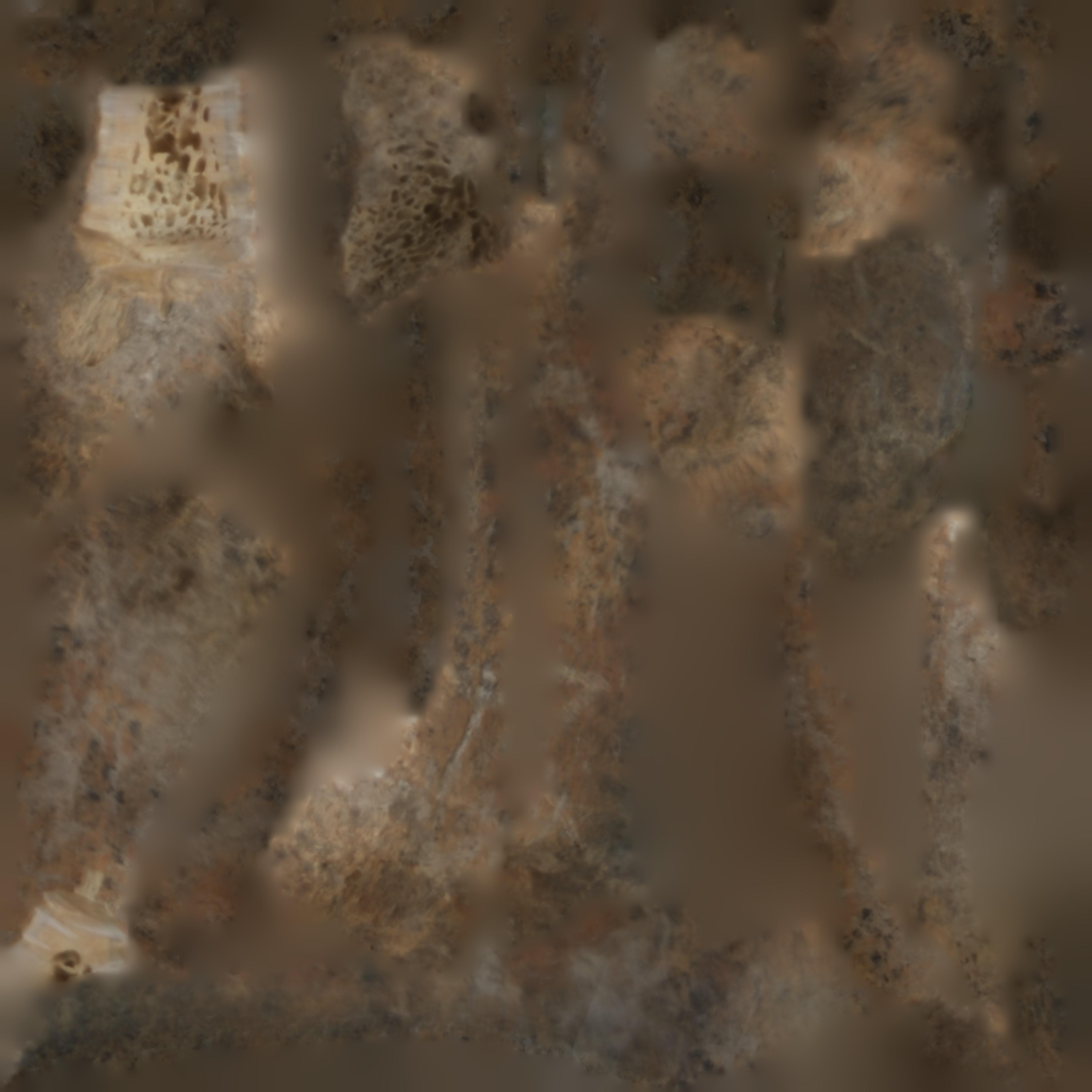

Supplement: Supplementary file 2 — Supplementary Datasets. [file 41598_2020_71572_MOESM2_ESM.zip › bearbone/BearBone_bearbone.jpg]
